# Revealing Missing Links in the Downsizing of the Photosystem II Antenna in Higher Plants Under Stress Conditions

**DOI:** 10.3390/antiox14121505

**Published:** 2025-12-15

**Authors:** Anatoly A. Nikolaev, Natalia N. Rudenko, Natalia S. Novichkova, Daria V. Vetoshkina, Maria M. Borisova-Mubarakshina

**Affiliations:** Institute of Basic Biological Problems RAS, Federal Research Center Pushchino Scientific Center for Biological Research of RAS, 142290 Pushchino, Moscow Region, Russia; nikolaevan@pbcras.ru (A.A.N.); rudenkon@pbcras.ru (N.N.R.); novichkova@pbcras.ru (N.S.N.); vetoshkina_d@pbcras.ru (D.V.V.)

**Keywords:** hydrogen peroxide, photosystem II antenna, plant acclimation, retrograde signaling, plastoquinone pool, serine protease

## Abstract

Chloroplast-to-nucleus ROS retrograde signaling is essential for acclimation of the photosynthetic apparatus to environmental stresses. One of the key mechanisms is the regulation of the photosystem II antenna size depending on light conditions and other environmental factors. However, the molecular components linking chloroplast redox status to nuclear gene regulation remain poorly defined. Here, we demonstrate that H_2_O_2_, generated in chloroplasts, in particular with involvement of the plastoquinone pool components, enhances the protease activity in the chloroplast envelope. As it is known, protease activity leads to the processing of the chloroplast envelope-bound transcription factor PTM, enabling its relocation to the nucleus, where it induces ABI4 expression. ABI4, in turn, represses transcription of *lhcb* genes, resulting in downsizing of the PS II antenna. Gene expression analysis confirms the coordinated upregulation of *ABI4*, and *PTM*, as well as metallo-*ASP* and serine *SPPA1* envelope proteases in high light. We further show that H_2_O_2_ at physiologically relevant concentrations specifically stimulates the serine protease activity, since this activation is inhibited by PMSF. Our findings indicate a link between redox changes in the plastoquinone pool and the H_2_O_2_ level in chloroplasts with protease-mediated signaling cascades. Therefore, the obtained data reveal the connection between chloroplast and nuclear control of photosynthetic light harvesting, highlighting a signaling strategy for the photosystem II antenna size regulation in higher plants.

## 1. Introduction

In the photosynthetic electron-transport chain (PETC) of higher plants, algae, and cyanobacteria, absorption of sunlight energy and subsequent photochemical conversion of energy are realized by PS II and PS I supercomplexes. Each photosystem comprises a core complex containing a reaction center (RC) and a light-harvesting complex, which are composed of pigment-protein complexes of varying composition.

PS II is the initial functional complex of PETC in thylakoid membranes. The structure of PS II was studied in detail and determined by X-ray diffraction analysis at a high resolution [1,2,3,4]. RC of PS II is formed by D1 (PsbA) and D2 (PsbD) proteins, which bind the redox cofactors and CP43 (PsbC), CP47 (PsbB) complexes containing chlorophyll *a*, carotenoids, and low molecular mass proteins [5]. The PS II antenna includes two parts: an inner antenna and an outer, or peripheral, antenna. CP43 and CP47 can be attributed to the internal antenna. In the peripheral antenna, three major PS II antenna proteins, Lhcb1, Lhcb2, and Lhcb3, are encoded by the *lhcb1*, *lhcb2*, and *lhcb3* nuclear genes, respectively; these proteins form predominantly heterotrimers: S-type (strongly bound to RC), M-type (moderately bound), and L-type (loosely bound) [6,7,8]. The S and L trimers are composed of two Lhcb1 proteins and one Lhcb2 protein. The M trimer consists of two Lhcb1 proteins and one Lhcb3 protein [9]. The three small monomeric proteins Lhcb4 (CP29), Lhcb5 (CP26), and Lhcb6 (CP24) are encoded by the *lhcb4*, *lhcb5*, and *lhcb6* nuclear genes [10]. The peripheral antenna, in addition to chlorophyll *a* and carotenoids, contains chlorophyll *b* as well.

Light-induced impairment of PS II predominantly arises from oxidative degradation of the D1 protein mediated by reactive oxygen species (ROS) [11,12]. Several mechanisms have been developed in plants to protect this photosystem (for rev. see [13]). One of these is regulation of the PS II antenna size, allowing the antenna cross-section and, therefore, the amount of energy transferred to RC to vary. The discovery of this mechanism can be summarized as follows. It was shown that higher plants and algae increase the PS II antenna size under shaded conditions and, conversely, decrease the PS II antenna size under high light conditions, thereby optimizing photosynthetic activity under both conditions [14,15]. Lindahl et al. [15] observed a decrease in the PS II antenna size by suppressing the biosynthesis of external Lhcb proteins under high light conditions lasting for more than three days. Therefore, this regulation was called a long-term acclimatory response of the photosynthetic apparatus to light conditions. Further, the selective proteolysis of Lhcb proteins has been shown to be triggered as early as 24 h after exposure to high light [16,17]. Among Lhcb proteins, the most significant decreases were observed for Lhcb1, Lhcb2, and Lhcb3, and for the minor polypeptide Lhcb6 [18,19]. The minimal antenna unit in high-light-adapted higher plants consists of Lhcb4, Lhcb5, and S-type trimers, in addition to the core complex and the inner antenna subunits of PS II [19,20,21]. The data of Frigerio et al. [21] and Borisova-Mubarakshina et al. [22] provided evidence that altering the intensity of biosynthesis of Lhcb proteins can be performed at both transcriptional and post-transcriptional levels, depending on the conditions used.

The transcriptional regulation of Lhcb protein biosynthesis, which is the most common mechanism controlling the content of antenna proteins in wild-type plants, is based on the so-called retrograde signaling from chloroplasts to the nucleus affecting the expression of nuclear genes (for rev. see [23]). One of the components of such regulation is the nuclear transcription factor ABI4 (ABSCISIC ACID INSENSITIVE 4) [24]. ABI4 can specifically bind to the promoters of stress-responsive genes, regulating their expression (for rev. see [25]). It has been shown that in Arabidopsis, the binding site of ABI4 in the *lhcb* gene promoters is the sequence -170 GGACCACAGTAG -159 in the cis-acting region of the promoter [26]. The activation of ABI4 leads to its interaction with the promoter region of *lhcb* genes, inhibiting *lhcb* gene expression and therefore leading to downsizing of the PS II antenna. A potential connection of ABI4 and the retrograde signaling, in particular for the PS II antenna size regulation, could be PTM, a chloroplast envelope-associated homeodomain transcription factor. This protein possesses transmembrane domains and is located in the chloroplast envelope. Sun et al. [27] showed that in response to treatments that initiate retrograde signals, including excess light conditions, PTM is modified and released from the plastid envelope into the cytoplasm with further accumulation in the nucleus, where it directly activates ABI4 gene expression. The soluble form of PTM is formed by proteolysis by a chloroplast membrane protease, resulting in the cleavage of its transmembrane domains [28]. Two potential candidates for such protease can be discussed according to the literature. The first is ASP (*At2g32480*), a light-regulated metalloprotease located on the inner membrane of the chloroplast envelope, and belongs to the conserved Site-2 Protease family of membrane metalloproteases. ASP has been shown to be essential for plastid development and a well-developed thylakoid system [29]. The second one is SPPA1 (signal peptide peptidase), an ATP-independent protease IV/endopeptidase of the serine type. SPPA1 (*At1g73990*) has previously been shown to be required for the antenna size regulation in *Synechocystis* [30]. SPPA1 is located both in thylakoid membranes and in the chloroplast envelope [31]. Therefore, the identity of the chloroplast-envelope protease responsible for proteolysis of PTM in strong light, and the mechanism of its activation, remain open questions.

The PS II antenna size has been shown to be regulated by the redox state of the plastoquinone pool (PQ pool) [21,32,33,34]. The PQ pool in general is considered as a central hub in plant redox metabolism. For example, Adamiec et al. [35] showed that the expression levels of 663 genes out of 24,000 studied were changed under high light, and the expression levels of 50 of them were changed in response to changes in the redox state of the PQ pool. The PQ pool redox state depends not only on the amount of energy absorbed but also on the ability of PS II to donate electrons and PS I to accept electrons. The oxidation of the reduced PQ, plastohydroquinone (PQH_2_), by the cytochrome *b*_6_/*f* complex is the rate-limiting step of photosynthesis. That is why the redox state of the PQ pool serves as a good indicator of the balance between light absorption and the rate of photosynthetic electron transfer.

Our recent studies demonstrated that the PQ pool components are involved in hydrogen peroxide (H_2_O_2_) formation in chloroplasts in the light. We have shown that H_2_O_2_ is produced not only as the result of the disproportionation of superoxide anion-radical (O_2_^•−^) catalyzed by superoxide dismutase in chloroplast stroma but also owing to the reaction of PQH_2_ with O_2_^•−^, in which O_2_^•−^ is neutralized to H_2_O_2_: PQH_2_ + O_2_^•−^ → PQ^•−^ (plastosemiquinone) + H_2_O_2_ [36,37,38,39]. H_2_O_2_ is the most important signal molecule among ROS; H_2_O_2_ formed in chloroplasts was found to activate early responses, including transcription factors and biosynthetic genes involved in the formation of second signaling messengers [40]. We previously proposed that PQ-pool-derived H_2_O_2_ can represent a molecular signal about the redox state of the PQ pool for retrograde signaling, and the progression of the described reaction can give insight into the molecular mechanism of both antioxidant and signaling functions of plastoquinone in plant cells [41,42,43]. Indeed, in the study of Borisova-Mubarakshina et al. [42], it has been experimentally proven that not the redox state of the PQ pool itself, but the amount of H_2_O_2_ formed in photosynthetic cells, plays a decisive role in regulating the PS II antenna size. However, the precise mechanism of H_2_O_2_ involvement in the PS II antenna size regulation remains to be elucidated.

We have previously shown that H_2_O_2_ molecules formed in chloroplasts diffuse through the chloroplast-envelope membrane into the cytoplasm, and the amount of H_2_O_2_ released from chloroplasts depended on light intensity, illumination time, and the activity of the chloroplast antioxidant system [44,45]. Moreover, according to the data of Biver et al. [46], some proteases can be activated by H_2_O_2_. Thus, we hypothesize that H_2_O_2_ formed by chloroplast components in the light, including components of the PQ pool, when diffusing through the chloroplast envelope, can activate a chloroplast-envelope protease and thereby trigger a retrograde regulatory pathway that ultimately leads to a decrease in the PS II antenna size. In the present study, we evaluated regulatory changes in the biosynthesis of components of the described PS II antenna size regulation mechanism in high light and revealed the interplay between the components involved with the attention given to the role of H_2_O_2_ in chloroplast-envelope protease(s) functioning. Thus, the results of the present study make it possible to reveal the missing links in the retrograde signaling pathway and present a general picture of this important acclimatory pathway in higher C3 plants.

## 2. Materials and Methods

### 2.1. Plant Material and Growth Conditions

*A. thaliana* plants of the Columbia ecotype (WT) were used. The seeds were sown in pots with soil and placed in a climatic chamber at a constant temperature of 18–20 °C and illumination of 100–110 μmol quanta m^−2^ s^−1^ (24 h). After seed germination, plants were grown under moderate illumination conditions of 100–110 μmol quanta m^−2^ s^−1^, 8 h day/16 h night (control plants). After 14–21 days, at the time of the formation of four true leaves, plants were transplanted into individual pots with a soil volume of 150 mL. For the experiments, 45-day-old plants were used. Then one part of plants was placed under 60–70 μmol quanta m^−2^ s^−1^ (8 h day/16 h night), the other part of plants was placed under 500 μmol quanta m^−2^ s^−1^ (8 h day/16 h night) for 9–12 days.

For the experiments with the constant high light treatment, Arabidopsis leaves were taken after 3, 24, 48, 72, and 144 h of constant illumination by 500 μmol quanta m^−2^ s^−1^ with no dark period for the measurements of the gene expression levels and after 24 h for the measurement of H_2_O_2_ level. Leaves from plants grown under 60–70 or 100–110 μmol quanta m^−2^ s^−1^ (8 h day/16 h night) were taken concurrently 144 h after the beginning of the experiment. Wild type plants of *Spinacia oleracea* were obtained from a local market (City Market, 74 Voroshilova Street, Serpukhov city, Moscow region, Russia). For experiments under increased illumination, spinach leaves were placed in water and kept under 500 μmol quanta m^−2^ s^−1^, 16 h day/8 h night for 4 days.

### 2.2. Measurement of Chlorophyll a Fluorescence

OJIP chlorophyll *a* fluorescence transients were measured in Arabidopsis leaves using a HandyPEA fluorometer (Hansatech, King’s Lynn, UK). Leaves were illuminated with 1 s red-light flashes (3000 µmol quanta m^−2^ s^−1^), and the OJIP curves were recorded. Plants were dark-adapted for 1.5–2 h prior to measurements. Photosynthetic parameters were calculated according to Tóth et al. [47] and Kalaji et al. [48].

The F_v_/F_m_ parameter represents the maximum quantum yield of PS II, where F_m_ is the maximal fluorescence when all PS II reaction centers are closed, and F_v_ is the variable fluorescence (F_v_ = F_m_ − F_0_, with F_0_ representing minimal fluorescence when all PS II reaction centers are open); ABS/RC = (M_0_/V_J_)(F_v_/F_m_) reflects the effective size of the PS II antenna, where M_0_ is the initial slope of the O-J phase of fluorescence growth (its value reflects the rate of closing of the PS II reaction centers); V_J_ = (F_J_ − F_0_)/F_v_ is the relative amplitude of the O-J phase (its value reflects the number of reaction centers that close when a flash of saturating light is applied, in relation to the total number of reaction centers); PI abs = (RC/ABS)(φPo/(1 − φPo))(ψ0/(1 − ψ0)) is the index of functional activity of PS II related to the absorbed energy, where φPo is the maximum quantum yield of the primary photochemical reaction, which indicates the probability of energy capture by the reaction centers of PS II, ψ0 is the value, which reflects the probability of electron transport beyond Q_A_^−^. Sm = Area/(F_m_ − F_0_) is the normalized area above the induction curve, reflecting the capacity of the pool of electron acceptors until complete reduction of Q_A_, where area is the area above the fluorescence induction curve.

### 2.3. Determination of Hydrogen Peroxide Content in Plant Leaves

The hydrogen peroxide (H_2_O_2_) content in plant leaves was determined using the luminol peroxidation reaction, as described by Cormier and Prichard [49]. Leaf samples (50–100 mg) were rapidly frozen in liquid nitrogen for 10 s, then placed in 0.4 mL of 2 M trichloroacetic acid, and mechanically homogenized. H_2_O_2_ was extracted by adding 3 mL of 0.05 M potassium-phosphate buffer at pH 8.5. To remove chlorophylls and carotenoids, the samples were incubated with 5% polyvinylpolypyrrolidone (PVPP) and then centrifuged at 10,000× *g* for 10 min. After centrifugation, the supernatant was collected, and 2 M KOH was added to denature proteins.

For the determination of H_2_O_2_ content, 1 mL of luminol solution (2.26 × 10^−4^ M) and peroxidase (1 × 10^−6^ M) were added to 50 μL of the obtained extract using a dispenser, and chemiluminescence was measured using a Lum-100 luminometer. A calibration curve was constructed using solutions with known concentrations of H_2_O_2_. Chemiluminescence was recorded with PowerGraph software, version 3.3.12.

To calculate the peroxide-dependent chemiluminescence, all samples were measured in the presence and absence of 300 units/mL of catalase. The H_2_O_2_ content was determined by subtracting the catalase-treated signal from the untreated signal.

### 2.4. Quantitative Reverse-Transcription PCR

Total RNA was extracted from frozen Arabidopsis or spinach leaves, using the R-Plants-50 kit (Biolabmix, Novosibirsk, Russia), and treated with DNase EM-250 (Biolabmix, Russia) to eliminate any genomic DNA contamination. Complementary DNA synthesis was performed using the reverse transcription kit OT-1 (Sintol, Moscow, Russia) with oligo (dT) as a primer. Quantitative reverse transcription polymerase chain reaction (qRT-PCR) was performed with qPCRmix-HS SYBR (Evrogen, Moscow, Russia), using primer sequences according to Table 1. The resulting qRT-PCR data were normalized to the ubiquitin-encoding genes of Arabidopsis or spinach, respectively (Table 1). The PCR reactions were carried out in a LightCycler 96 Instrument (Roche Diagnostics, Basel, Switzerland).

### 2.5. Isolation of Envelope

To study the effect of hydrogen peroxide on the activity of chloroplast-envelope proteases, the method of Douce et al. [50] was modified. Spinach leaves from the local market were used to isolate chloroplasts. Spinach leaves were homogenized, and the leaf homogenate was filtered through a nylon filter. Chloroplasts were purified using a Percoll gradient. The chloroplast fraction was collected, washed, and then used to obtain the envelope. For “swelling”, the chloroplasts were placed in a medium with a low sucrose concentration (0.03 M) and incubated at 4 °C for 5 min. The resulting chloroplast fraction of about 5 mL was applied to a sucrose gradient (20.5 and 31.8%, 8 mL each) and centrifuged at 72,000× *g* for 1 h. The fraction of chloroplast envelope was collected from two boundary layers with different sucrose concentrations (the thylakoids were precipitated). The resulting fraction of chloroplast envelope was used for the experiments to assess the activity of the envelope proteases.

### 2.6. Evaluation of the Chloroplast-Envelope Protease Activity

Protease activity was determined spectrophotometrically by measuring the amount of tyrosine released from casein. The fractions of chloroplast envelope were incubated for 1 h at 45 °C in 0.65% casein (CDH Fine Chemical, Daryaganj, Delhi, India) in 0.1 M phosphate buffer (pH 7.5) in the presence of 1% n-dodecyl β-D-maltoside (Macklin Inc., Shanghai, China) to increase the availability of proteases to casein. H_2_O_2_ was added to the samples in concentrations of 25, 50, 100, 250, 500, and 1500 μM in the presence and absence of 4 mM phenylmethylsulfonyl fluoride, PMSF (Macklin Inc., Shanghai, China), as an inhibitor of the serine protease activity. PMSF was added to the envelope samples 30 min before the reaction. The reaction was stopped by adding 5% trichloroacetic acid, followed by centrifugation for 15 min at 3000× *g*. After removing the precipitate by filtration through filter paper, the absorbance of the filtrates was determined at 280 nm using a spectrophotometer (Hitachi, Hitachi-shi, Ibaraki Prefecture, Japan). Protease activity was expressed as mg of tyrosine released per mg of total chloroplast-envelope protein per hour, using a standard calibration curve prepared with L-tyrosine. Protein concentration was determined using the Bradford assay.

### 2.7. Immunoblotting

Total protein extraction from *A. thaliana* leaves was done using the method of Conlon and Salter [51]. The chlorophyll content was determined in 96% ethanol extracts [52]. Protein samples obtained were separated using denaturing electrophoresis in 16% polyacrylamide gels following the protocol of Schägger & von Jagow [53], in a Mini-PROTEAN Cell (BioRad, Hercules, CA, USA). Protein extracts were diluted in a loading buffer containing 200 mM Tris (pH 6.8), 2% (*w*/*v*) SDS, 0.005% (*w*/*v*) bromophenol blue, and 5% (*w*/*v*) dithiothreitol. Samples were heated to 95 °C for 5 min and centrifuged at 12,000× *g* for 5 min in a MiniSpin centrifuge (Eppendorf, Hamburg, Germany). The supernatants were then loaded onto gels in amounts corresponding to 0.5 or 1.0 μg of chlorophyll per lane. Protein Marker 10–180 kDa (Servicebio, Wuhan, China) was used for estimation of the protein molecular masses.

After electrophoresis, proteins were transferred from the gels to PVDF membranes (BioRad) using a Mini Trans-Blot Cell (BioRad, Hercules, CA, USA). The membranes were then incubated in Tris-buffered saline (TBS, BioRad, Hercules, CA, USA) containing 20 mM Tris (pH 7.6) and 150 mM NaCl for 5 min, followed by blocking for 60 min in TBS containing 2% (*w*/*v*) nonfat dried milk. The membranes were then incubated with primary polyclonal rabbit antibodies against Lhcb1, Lhcb2, and D1 proteins (AS01004, AS132705, and AS01010, respectively, Agrisera Co., Vännäs, Sweden), washed in TBS, and incubated with secondary goat anti-rabbit IgG antibodies (Agrisera, Vännäs, Sweden). Protein bands were visualized using an alkaline phosphatase conjugate substrate kit (BioRad). Membranes were scanned using a transmission flatbed scanner (Epson V700, Epson, Shinjuku, Tokyo, Japan) in a 16-bit per pixel high dynamic range mode.

Densitometric analysis of the Western blot results was performed to quantify the protein content in each lane by measuring the integrated optical density (IOD) of the protein bands using ImageJ software, version 1.54 and OriginPro software, version 2021.

Immunoblotting was also performed with the proteins of chloroplast envelope. Protein extracts of chloroplast envelope were prepared, and denaturing electrophoresis followed by immunoblotting was done as described above. Immunoblotting was performed with antibodies Anti-Toc75 (AS08 351, Agrisera, Vännäs, Sweden) for detecting the chloroplastic POTRA domain 3 Protein TOC75-3 (chloroplast outer envelope membrane protein marker).

### 2.8. Statistical Analysis

Statistical analysis was conducted using OriginPro software. ANOVA was performed, followed by post hoc comparison of means using the Holm–Bonferroni and Wilcoxon tests. Statistical significance was assessed at the *p* < 0.05, <0.01, and <0.001 levels.

## 3. Results

### 3.1. Effect of Increased Illumination on the Photosynthetic Characteristics of Intact Leaves

**Figure 1:**
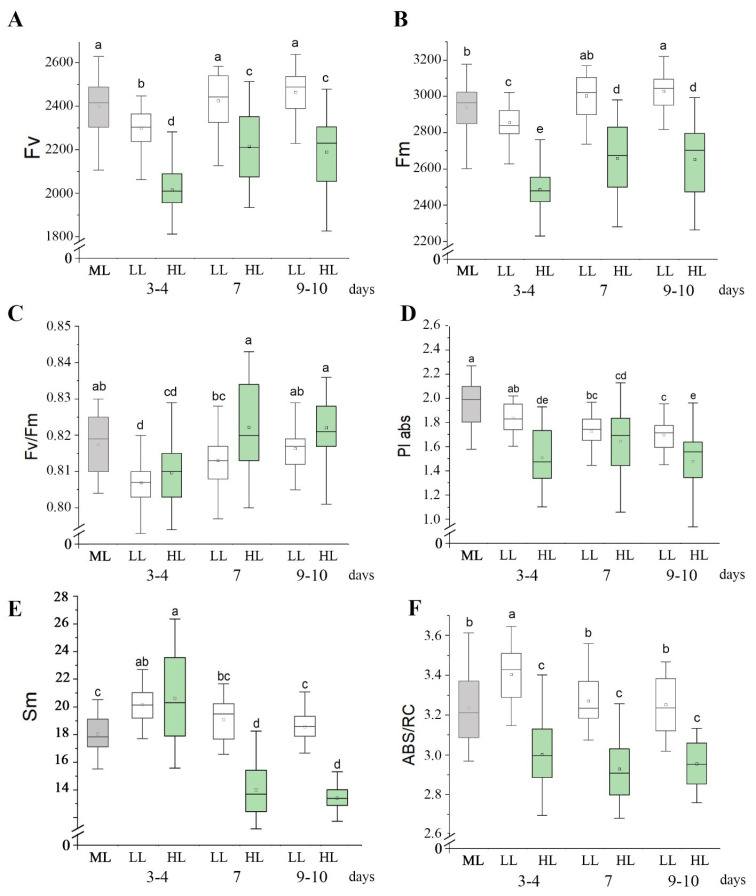
Effect of LL and HL on photosynthetic parameters in *Arabidopsis* leaves, as determined by OJIP curve measurements. F_v_ is the variable fluorescence of chlorophyll *a* of PS II (**A**); F_m_ is the maximal chlorophyll *a* fluorescence (**B**); F_v_/F_m_ is the maximum quantum yield of PS II (**C**); PI_ABS_ is the parameter of functional activity of PS II (**D**). Sm is the relative oxidation state of the PQ pool (**E**); ABS/RC is the effective antenna size of PS II (**F**). Grey boxes are the control plants (ML, grown at 100–110 μmol quanta m^−2^ s^−1^), white boxes are the plants adapted to low light (LL, grown at 60–70 μmol quanta m^−2^ s^−1^), green boxes are the plants adapted to high light (HL, 500 μmol quanta m^−2^ s^−1^). Along the *x*-axis, the number of days from the beginning of the experiment is given. For each time point, parameters were measured for 30 leaves. The boxes represent the interquartile range (IQR), encompassing the middle 50% of the data; the mean values are indicated by a square inside the boxes, and the horizontal line within each box marks the median. Different letters above the bars indicate significant differences between variants with *p* < 0.05 as determined by ANOVA, followed by a Holm-Bonferroni test.

The influence of high light (HL) and low light (LL) on photosynthesis was studied using measurements of high-resolution OJIP chlorophyll *a* fluorescence transients (OJIP curves). Based on the OJIP curves, the parameters characterizing the functioning of the photosynthetic apparatus in Arabidopsis plants were estimated (Figure 1).

Figure 1 shows the time dependence of the HL and LL effect on photosynthetic parameters of Arabidopsis plants. Three-four days after the beginning of the experiment there was a slight but statistically significant decrease in the variable (F_v_) and maximal (F_m_) chlorophyll *a* fluorescence levels (Figure 1A,B), maximum quantum yield of PS II (F_v_/F_m_) and the parameter of functional activity of PS II (PI_ABS_) (Figure 1C,D), for both LL and HL plants compared to control plants grown under moderate light intensity (ML, see Section 2). After 7 days under the changed light conditions, the parameters F_v_, F_m_, and F_v_/F_m_ began to increase, and in LL plants they became the same as in control ML plants (Figure 1A–C). By 9–10 days of adapting to HL F_v_/F_m_ parameter also reached the initial values (Figure 1C). The recovery of F_v_/F_m_ indicates successful adaptation of photosynthetic apparatus to both LL and HL conditions by the end of the experiment. At the same time, F_v_ and F_m_ in HL plants remained lower than in control plants (Figure 1A,B) and PI_ABS_ after its decrease on day 3–4 in both LL and HL plants to 9–10 days remained lower compared to its initial values (Figure 1D).

Sm parameter is proportional to the total number of accessible electron carriers until full reduction of Q_A_ centers, indicating a relative oxidation state of the PQ pool. Since the Sm value is affected not only by the redox state of the PQ pool, but also by the number of inactive centers of PS II (Q_B_-non-reducing centers), it can be used to assess the PQ pool state only if the F_v_/F_m_ value differs insignificantly between variants used [54,55]. That was observed in our experiments since F_v_/F_m_ was not lower than 0.8 in all variants during the whole experiment (Figure 1C,D). The observed F_v_/F_m_ changes indicate the absence of inhibitory effect of the light used, allowing the PQ pool redox state to be estimated. The Sm parameter was significantly higher by the 3rd–4th day of LL and HL treatments, indicating a lower reduction level of the PQ pool under these conditions compared to ML plants. However, by day 7 of the experiment, the Sm value in LL plants no longer differed from that in ML plants, while in HL-treated plants it was significantly lower, reflecting a higher reduction level of the PQ pool in HL compared to ML and LL plants (Figure 1E).

### 3.2. Adjustment of PS II Antenna Size at Different Plant Illumination Levels

To analyze the size of the PS II antenna under the studied conditions, the ABS/RC parameter was evaluated (Figure 1F). ABS/RC parameter characterizes the energy flux absorbed by one active reaction center, which indirectly reflects the effective PS II antenna size. This parameter increased in LL plants and decreased in HL plants compared to control, ML plants, by 3rd–4th days of adaptation (Figure 1F). After that and till the end of the experiment, ABS/RC remained reduced in HL plants, whereas in LL plants it returned to the initial, control values (Figure 1F). Thus, HL treatment results in a decrease in ABS/RC compared to LL and ML, reflecting a downsizing of the functional PS II antenna under HL conditions.

Further, Chl *a*/Chl *b* was evaluated after 10 days of HL exposure; changes of this parameter serve as additional indicator of the PS II antenna size alterations given that the core antenna subunits of PS II, CP47 and CP43, in addition to carotenoids, bind Chl *a* only, whereas the outer antenna complexes bind both Chl *a* and Chl *b* (see Introduction). According to our measurements, Chl *a*/Chl *b* in leaves increased from 2.44 ± 0.06 in LL plants to 2.69 ± 0.08 in HL plants. Thus, based on the Chl *a*/Chl *b* measurements, the downsizing of the PS II antenna was confirmed under the studied HL conditions, which is in agreement with the data of [15,18,19].

**Figure 2:**
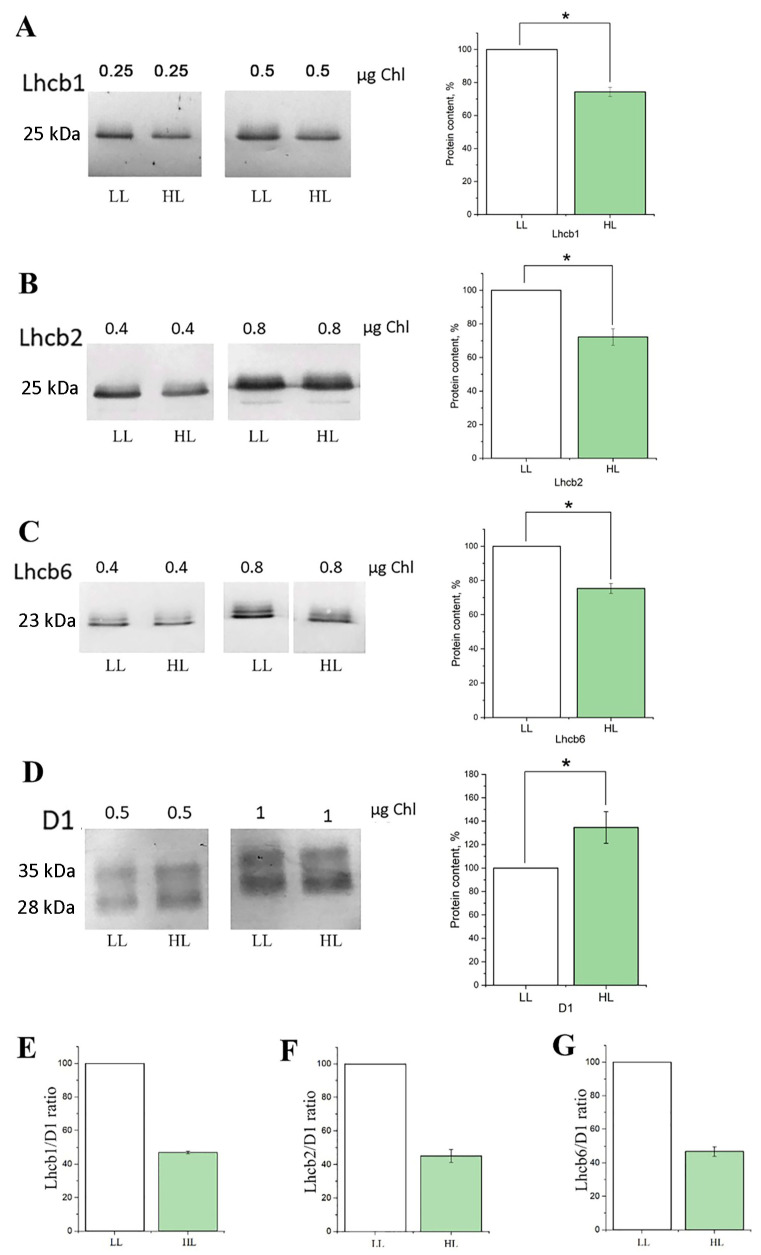
Typical immunoblots obtained after denaturing electrophoresis of the total protein leaf extracts with antibodies against Lhcb1 (**A**), Lhcb2 (**B**), Lhcb6 (**C**), and D1 (**D**) proteins. The optical densities of Lhcb1, Lhcb2, Lhcb6, and D1 protein bands are shown as column bars on the right side of the figure. The optical density ratios of Lhcb1, Lhcb2, and Lhcb6 normalized to D1 are shown as Lhcb1/D1 (**E**), Lhcb2/D1 (**F**) and Lhcb6/D1 (**G**). White boxes—control plants (LL, 60–70 μmol quanta m^−2^ s^−1^), green boxes—plants transferred to high light conditions for 9–10 days (HL, 500 μmol quanta m^−2^ s^−1^). Results are presented as the mean of three independent measurements. Error bars represent the standard error of the mean. The asterisk indicates significant differences determined by ANOVA, followed by a Holm-Bonferroni test: *p* < 0.05. Full immunoblot membranes indicating the molecular weight of the marker proteins are in the Supplementary Figure S1A–D.

Also, after 10 days of HL, total protein extracts were isolated from LL and HL plants. The content of PS II outer antenna proteins, such as Lhcb1, Lhcb2, and Lhcb6 (Figure 2), was quantified. The amount of these proteins in leaves of plants exposed for 10 days to HL was lower than in plants exposed to LL conditions (Figure 2A–C). In addition, the abundance of the major PS II reaction center protein, D1, was estimated. No decrease of D1 protein was detected under increased light conditions, compared to LL conditions (Figure 2D), which is in agreement with the F_v_/F_m_ data presented in Figure 1A–C. Even a slight increase in the amount of this protein was detected. Thus, selected HL conditions had no inhibitory effect on the activity of PS II and, therefore, the whole photosynthetic electron-transport chain. Increasing the light intensity to 1100 μmol quanta m^−2^ s^−1^ resulted in a pronounced decrease in the D1 protein level by the 7th day (Figure 2D) without further recovery by 10th day. That was the reason why the light intensity of 500 μmol m^−2^ s^−1^ for HL treatment was chosen in our experiments.

To verify changes in the content of PS II antenna proteins related to the antenna size alterations under LL and HL conditions, the content of Lhcb1, Lhcb2, and Lhcb6 proteins was normalized to the content of D1 protein. Figure 2E–G show the pronounced decrease in the levels of the analyzed PS II antenna proteins after 10 days of HL compared to those in LL, reflecting the PS II antenna downsizing. These results are consistent with the above presented results obtained by analyzing ABS/RC and Chl *a*/Chl *b* parameters, as well as with the literature data (see above).

**Figure 3:**
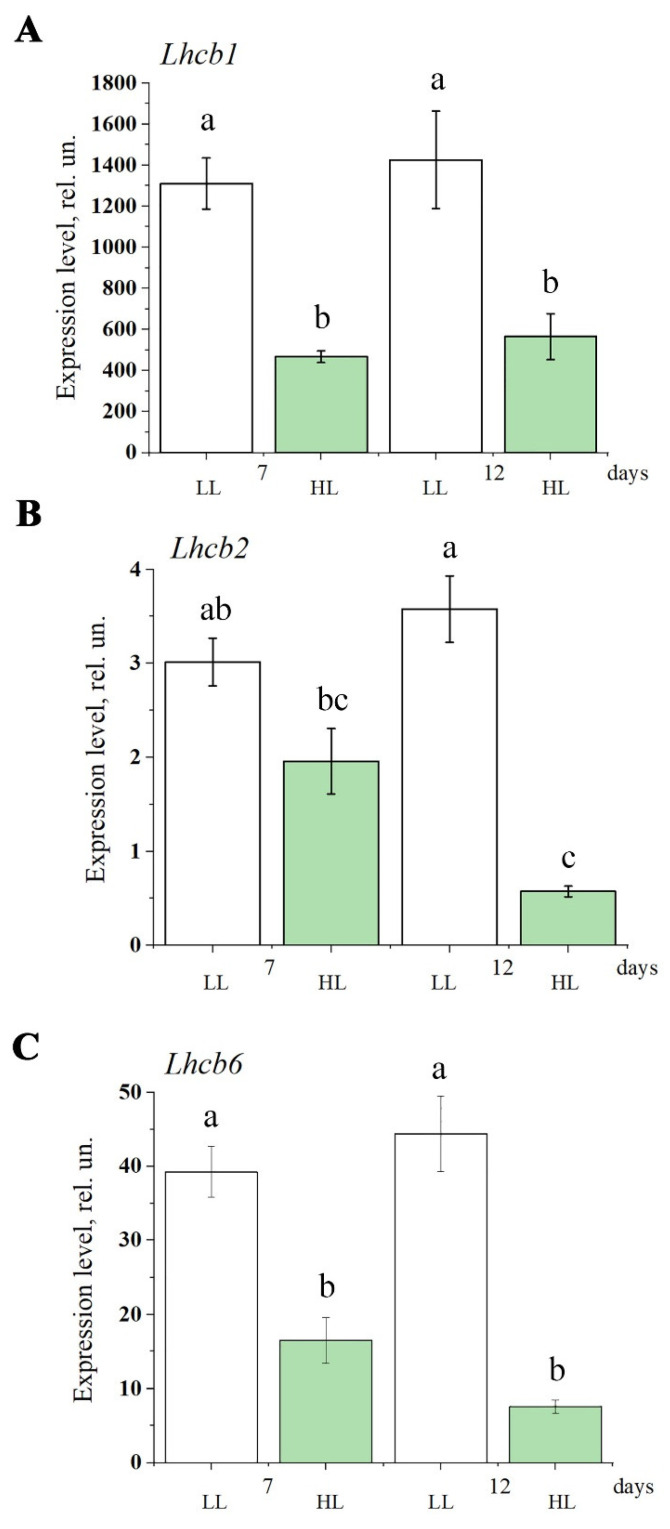
The effect of light intensity on the expression level of *lhcb1* (**A**), *lhcb2* (**B**), and *lhcb6* (**C**) genes in the leaves of Arabidopsis plants. White boxes are the low light plants (LL, 60–70 μmol quanta m^−2^ s^−1^), green boxes are the plants transferred to high light conditions (HL, 500 μmol quanta m^−2^ s^−1^). Along the *x*-axis, the number of days from the beginning of the experiment is given. Error bars represent the standard error of the mean. Different letters above the bars indicate significant differences between variants with *p* < 0.05 as determined by ANOVA, followed by a Holm-Bonferroni test.

During the acclimation to HL, the content of the *lhcb1*, *lhcb2*, and *lhcb6* transcripts decreased compared to LL (Figure 3). At the same time, a decrease in *lhcb1* and *lhcb6* expression has been detected on the 7th day of HL adaptation, with no further changes by the 12th day; the diminishing of *lhcb2* expression was more pronounced by the 12th day compared to the 7th day.

### 3.3. Enhancing the Signal Transmission Pathway for Regulation of the PS II Antenna Size in High Light

The rearrangement of the PS II antenna size, which represents a long-term mechanism of adaptation of higher plants to light intensity changes, is regulated by the redox state of the PQ pool [33,56]. This is also what we observed according to the data presented above: the higher reduced level of the PQ pool, followed by the PS II antenna size downsizing, was detected after 3rd–4th days of HL. Since a highly reduced PQ pool should be accompanied by the enhanced H_2_O_2_ production for the studied retrograde signaling pathway (see Introduction), we further evaluated the H_2_O_2_ level in leaves adapted to either LL or HL for 10 days.

**Figure 4:**
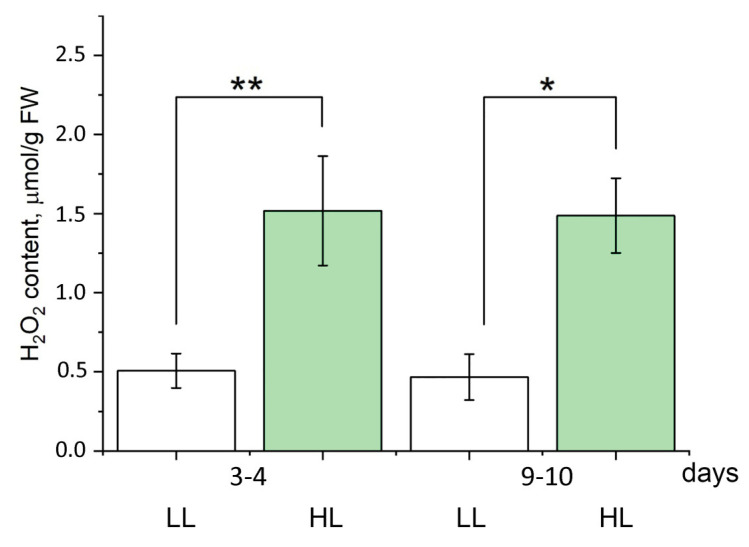
Influence of HL (500 μmol quanta m^−2^ s^−1^) treatment on H_2_O_2_ content in Arabidopsis leaves at 3rd–4th days and 9th–10th days. White boxes are control plants (LL, 60–70 μmol quanta m^−2^ s^−1^), green boxes are the plants transferred to high light conditions (HL, 500 μmol quanta m^−2^ s^−1^). Along the *x*-axis, the number of days from the beginning of the experiment is given. Results are presented as the mean of three independent measurements. Error bars represent the standard error of the mean. Significant differences determined by ANOVA, followed by a Holm-Bonferroni test: * *p* < 0.05, ** *p* < 0.01.

As shown in Figure 4, on the 4th day after HL treatment, the H_2_O_2_ level in Arabidopsis leaves had already increased. These data show that an increase in the PQ pool reduction level (Figure 1E) results in an intensification in H_2_O_2_ formation. Increase in hydrogen peroxide content triggers a signaling pathway leading to changes in the expression of nuclear genes encoding PS II antenna proteins, and consequently to a decrease in the PS II antenna size.

**Figure 5:**
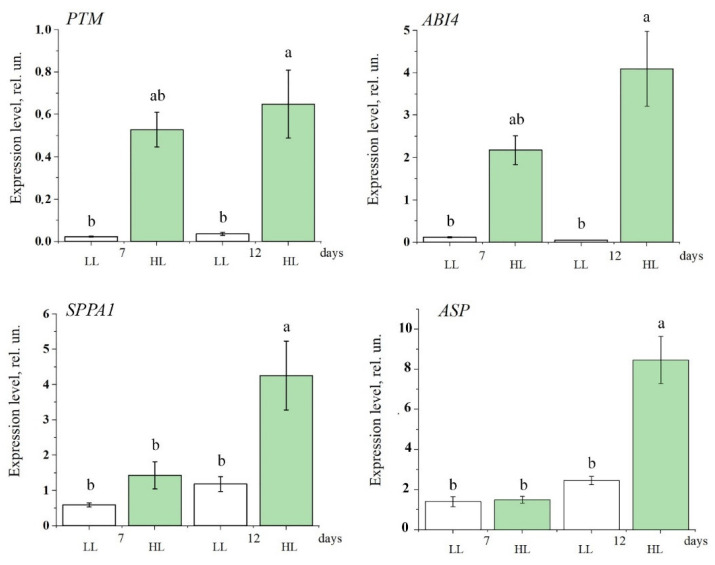
The effect of light intensity on the expression level of genes encoding transcription factors PTM (*At5g35210*) and ABI4 (*At2g40220*), SPPA1 serine protease (*At1g73990*), and ASP metalloprotease (*At2g32480*), in the leaves of Arabidopsis plants. White boxes are plants transferred to low light conditions (LL, 60–70 μmol quanta m^−2^ s^−1^), green boxes are plants transferred to high light conditions for 7 and 12 days (HL, 500 μmol quanta m^−2^ s^−1^). Error bars represent the standard error of the mean. Different letters above the bars indicate significant differences between variants with *p* < 0.05 as determined by ANOVA, followed by a Holm-Bonferroni test.

Since the expression of genes encoding Lhcb proteins is downregulated due to binding of ABI4 to the promoter sequences of these genes [24,26,57] (see Section 1), we measured the expression of the ABI4 (*At2g40220*) and PTM (*At5g35210*) genes over the course of adaptation to HL (Figure 5). It was found that increased illumination led to an enhancement of mRNA levels of *ABI4* and *PTM* (Figure 5). It should be noted that a very low expression of *ABI4* and *PTM* mRNA was observed under LL conditions, which is in accordance with literature data showing that the expression of these factors is barely detectable under normal growing conditions [58].

Given the cooperative involvement of a chloroplast-envelope protease, together with ABI4 and PTM, in the regulation of PS II antenna size, we evaluated the effect of HL on the expression of the genes encoding known envelope proteases, a metalloprotease ASP, and a serine protease SPPA1 under the same conditions. qRT-PCR analysis showed that HL exposure, as in the case of *ABI4* and *PTM*, resulted in an increase in both *ASP* and *SPPA1* mRNA levels (Figure 5), clearly demonstrating activation of the studied signaling pathway for PS II antenna downsizing under HL conditions.

### 3.4. The Effect of Continuous High Light on Expression Levels of the Genes Encoding Proteins Involved in PS II Antenna Size Regulation and H_2_O_2_ Content

**Figure 6:**
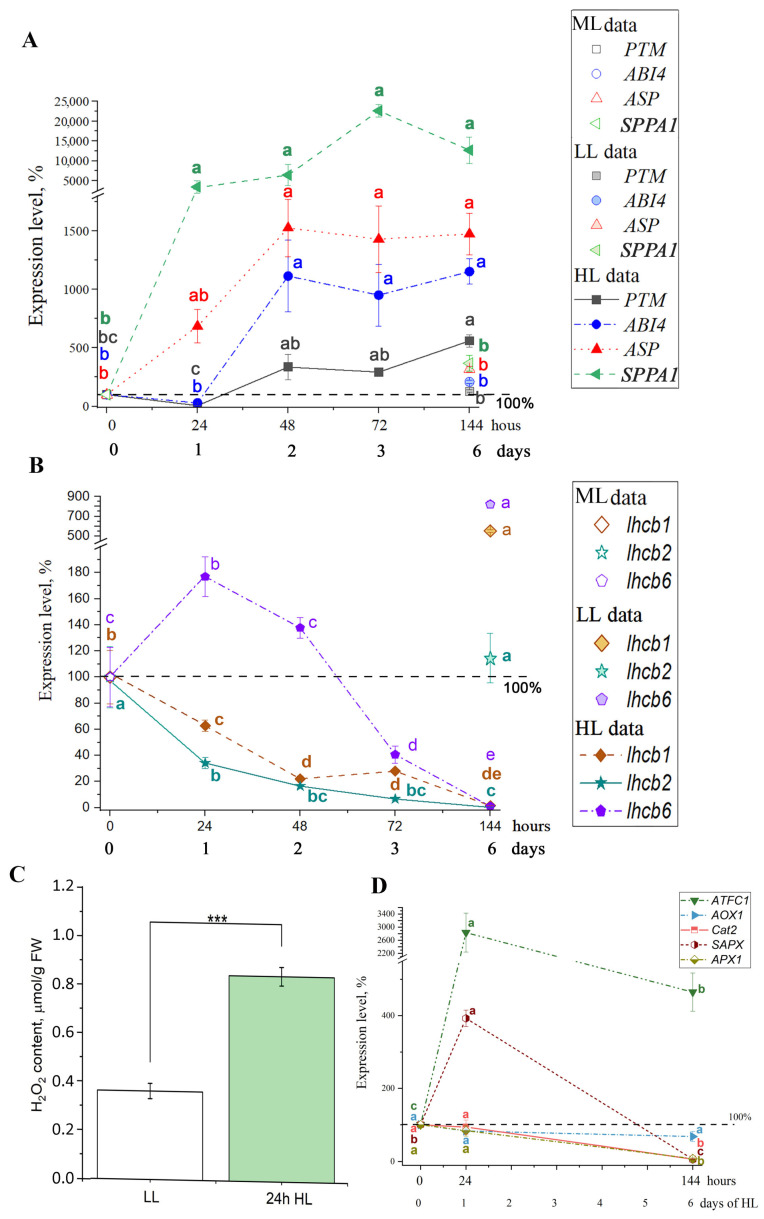
The effect of continuous high light intensity on the expression level of genes encoding SPPA1 serine protease (*At1g73990*) and ASP metalloprotease (*At2g32480*), transcription factors PTM (*At5g35210*) and ABI4 (*At2g40220*) (**A**); *lhcb1* (*At1g29930*), *lhcb2* (*At2g05070*) and *lhcb6* (*At1g15820*) (**B**); *ATFC1* gene (*At5g26030*), encoding ferrochelatase I located in plastids, *AOX1* gene (*At3g22370*)*,* encoding an isoform of alternative oxidase located in mitochondria, *APX1 (At1g07890)* gene, encoding a cytosolic ascorbate peroxidase 1, *SAPX* gene *(At4g08390*)*,* encoding chloroplast stromal ascorbate peroxidase, and *Cat2* gene (*At4g35090*) encoding a peroxisomal catalase 2 (**D**). 100% is the expression of 1.40 for *ASP*, of 0.28 for *PTM*, of 0.88 for *ABI4*, of 693.18 for *lhcb1*, of 77.12 for *lhcb2*, of 29.90 for *lhcb6,* of 1.27 for *ATFC1* (At1g07890), encoding cytosolic ascorbate peroxidase APX1, of 0.30 for *AOX1*, of 0.55 for *APX1*, of 0.58 for *SAPX*, and of 11.21 for *Cat2* at 0 h in the control, medium light conditions (90–100 μmol quanta m^−2^ s^−1^, 8/16 h photoperiod). Different letters within the bars indicate significant differences between variants with *p* < 0.05 as determined by Holm-Bonferroni test; the colors of the letters correspond to the color of the graphs for each individual gene. (**C**) H_2_O_2_ level under LL and after 24 h under HL in the leaves of Arabidopsis plants, LL—plants grown at low light intensity (60–70 μmol quanta m^−2^ s^−1^, 8/16 h photoperiod), HL—plants transferred to high light conditions (HL, 500 μmol quanta m^−2^ s^−1^, 24 h photoperiod). Error bars represent the standard error of the mean. Significant differences determined by ANOVA, followed by a Holm-Bonferroni test: *** *p* < 0.001.

In order to assess how fast the expression levels of genes encoding both chloroplast-envelope proteases as well as ABI4 and PTM increase, and how fast the expression levels of genes encoding the proteins of Lhcb decrease, we analyzed the changes in mRNA levels of the corresponding genes under constant illumination. The expression of genes encoding ASP and SPPA1 was considerably higher by 24 h of HL exposure; the expression of genes encoding ABI4 and PTM transcription factors increased after 24 and 48 h from the onset of HL illumination (Figure 6A), and remained high for up to 144 h under HL.

The inhibitory effect of HL on the expression of *lhcb1* and *lhcb2* genes has also manifested after 24 h under HL conditions. The expression levels of *lhcb1* and *lhcb2* gradually decreased with exposure to HL, and after 144 h, these levels constituted only 1.5% and 0.15% of the initial values of ML plants, respectively (Figure 6B). The expression level of the *lhcb6* gene, in contrast, increased approximately twofold after 24 h under HL. It started to decrease after 48 h of HL, approaching the ML level, but by 72 h of HL, it constituted only 40% of the initial ML value. By 144 h of HL, it constituted only 0.3 r.u., that is, about 1% of initial value in ML conditions, as was observed for the *lhcb1* and *lhcb2* transcripts (Figure 6B). After 144 h in LL conditions, the level of *lhcb2* gene expression was close to the initial values, while the expression levels of *lhcb1* and *lhcb6* genes increased by 5.5- and 8-fold, respectively, compared to ML (Figure 6B).

Since 24 h of constant HL illumination is the crossover point for changes in the expression of most of the genes studied, we estimated the level of H_2_O_2_ in plant leaves after 24 h under these conditions (Figure 6C). The H_2_O_2_ level increased approximately twofold under HL conditions compared to LL conditions. Thus, we observed a significant increase in H_2_O_2_ content after 24 h of HL, followed by an increase in *ABI4*, *PTM*, *SPPA1,* and *ASP* expression. At the same time, we observed a decrease in the expression of *lhcb1* and *lhcb2* genes encoding the main proteins of the PS II antenna. These data provide additional evidence that H_2_O_2_ is the signal that is transmitted to decrease the size of the light-harvesting PS II antenna via downregulation of *lhcb* genes.

H_2_O_2_ can be generated not only in chloroplasts but also in several other subcellular compartments, including peroxisomes, mitochondria, and the plasma membrane. Chloroplasts have been identified as the predominant source of enhanced H_2_O_2_ production under high light conditions [44,59]. Considering that other subcellular sources may still contribute to the overall H_2_O_2_ pool even in strong light, we additionally examined the expression of several marker genes associated with distinct organelles: *ATFC1* (*At5g26030*), encoding plastid ferrochelatase I, a recognized marker of chloroplast retrograde signaling; *AOX1* (*At3g22370*), encoding the mitochondrial alternative oxidase (AOX); ascorbate peroxidases (APX): *APX1* (*At1g07890*), encoding cytosolic ascorbate peroxidase 1; *SAPX* (*At4g08390*), encoding the stromal isoform of chloroplast ascorbate peroxidase; and *Cat2* (*At4g35090*), encoding peroxisomal catalase. A significant increase in the expression of *SAPX* and *FC1* was detected after 1 day of exposure to high light conditions (Figure 6D). In contrast, the transcript levels of *APX1* and *Cat2* do not exhibit an upregulation under high light conditions (Figure 6D). No upregulation was also observed for *AOX1*, a marker of mitochondrial retrograde signaling. Taken together, these transcriptional responses reflect the particular contribution of chloroplast-derived H_2_O_2_ to the studied cellular response under the conditions used.

### 3.5. The Effect of Hydrogen Peroxide on the Protease Activity of Isolated Chloroplast Envelope

To test our hypothesis that H_2_O_2_ formed by chloroplast components in the light can activate the chloroplast-envelope protease activity, we isolated chloroplast envelopes from spinach (*S. oleracea*) leaves. Spinach was chosen for envelope isolation since this procedure requires a large amount of biomass, which is difficult to obtain from Arabidopsis leaves.

**Figure 7:**
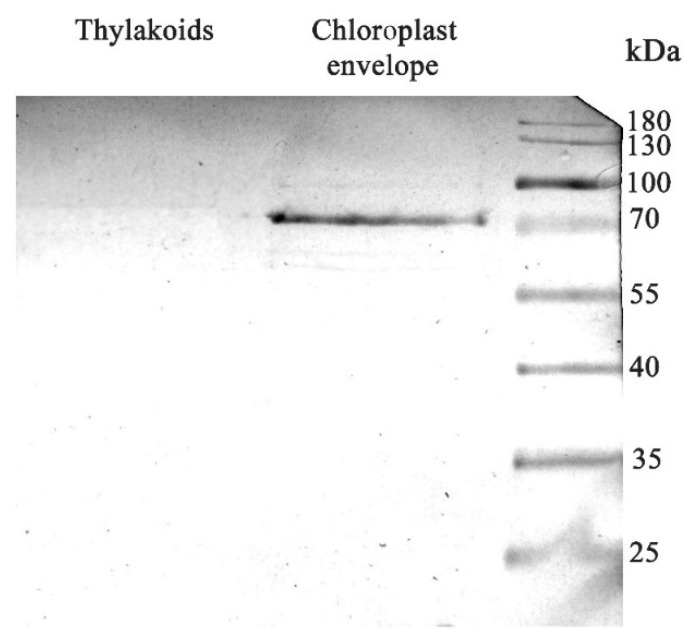
Immunoblotting with antibodies against chloroplast-envelope TOC-75 protein after denaturing electrophoresis of the chloroplast-envelope proteins isolated from *S. oleracea* leaves. Molecular mass markers with indicated molecular masses of proteins are in the right lane. Each gel line contained 325 μg of protein. Full immunoblot membrane is in the Supplementary Figure S1E.

Western blot analysis with antibodies against TOC-75 protein (Figure 7), a component of the translocon complex located on the outer membrane of the chloroplast envelope [60], was performed. Immunoblotting showed the presence of a TOC-75, confirming that the isolated chloroplast fraction corresponds to the chloroplast envelope. Western blot analysis with antibodies against TOC-75 protein was also performed with isolated thylakoids as a negative control. No band corresponding to 75 kDa was observed, confirming the purity of the obtained chloroplast envelope.

**Figure 8:**
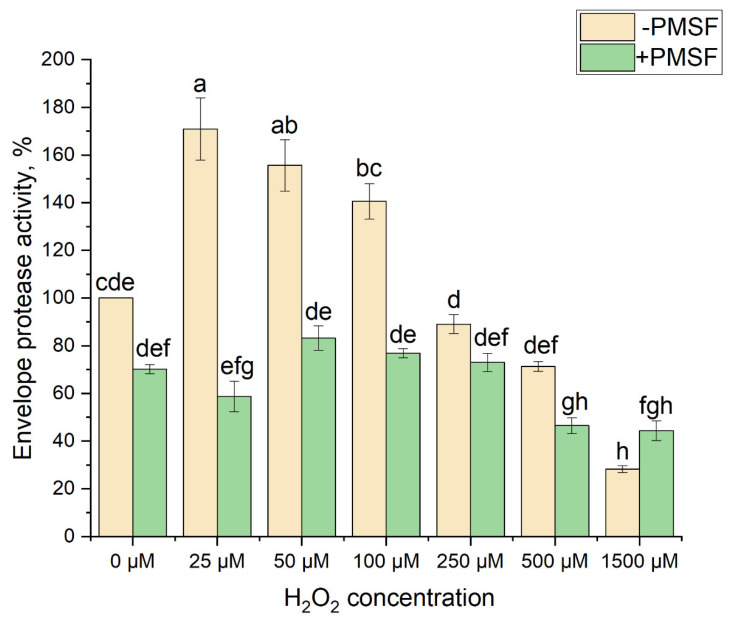
The effect of H_2_O_2_ on the activity of proteases of chloroplast envelope from *S. oleracea*. Brown bars represent protease activity measured in the absence of the serine protease inhibitor PMSF; green bars represent protease activity measured in the presence of the PMSF. Protease activity was expressed as mg of tyrosine released per mg of total chloroplast-envelope protein per hour. Results are presented as the mean of three independent measurements. Error bars represent the standard error of the mean. Different letters above the bars indicate significant differences between variants with *p* < 0.05 as determined by ANOVA, followed by a Holm-Bonferroni test.

The total activity of proteases located in the chloroplast envelope was estimated (Figure 8). Casein was used as a substrate for protease activity determination (see Section 2). When protease(s) digested casein, the amino acid tyrosine is released along with other amino acids and peptide fragments. The more tyrosine is released from casein, the higher the protease activity. The total protease activity was, on average, about 0.08 optical units (this value was taken as 100% corresponding to 1.2 mg Tyr per 1 mg envelope protein per hour). The total protease activity increased with addition of H_2_O_2_ at a concentration of up to 100 μM; the greatest increase of activity, 171 ± 13% of the initial one, was observed in the presence of 25 μM H_2_O_2_. When the H_2_O_2_ concentration was increased to 50 μM, the increase in protease activity was 156 ± 11%, and at 100 μM H_2_O_2_ concentration, 141 ± 13%. Almost no increase in the envelope protease activity was detected when using 250 μM H_2_O_2_. Adding H_2_O_2_ at concentrations higher than 500 μM, on the contrary, caused inhibition of the protease activity (Figure 8).

In order to identify the nature of the protease, the activity of which is enhanced by H_2_O_2_ at low concentration, an inhibitory analysis was applied for further experiments. Taking into account that two main candidates, SPPA1 serine protease and ASP metalloprotease, are considered (see Introduction), we used PMSF, which is a specific inhibitor of serine protease activity. Firstly, the addition of PMSF in the absence and presence of H_2_O_2_ at different concentrations made it possible to establish the contribution of serine proteases to the total protease activity of the chloroplast membrane fraction. The addition of PMSF decreased the total protease activity of the envelope preparations by approximately 25%. The remaining 75% of the protease activity is apparently due to the presence of proteases of different classes other than serine ones in the chloroplast envelope. Addition of PMSF to the preparations incubated in the presence of H_2_O_2_ completely abolished the stimulating effect of H_2_O_2_ at concentrations of 25–100 μM (Figure 8), showing the specificity of H_2_O_2_-dependent activation of the serine protease functioning. An inhibitory effect of H_2_O_2_ at higher concentrations was similar to the inhibitory effect of hydrogen peroxide in the absence of PMSF. These results further substantiate that the activation observed under H_2_O_2_ treatment is a specific and regulated process rather than a nonspecific oxidative effect. This provides direct evidence that H_2_O_2_ does not act merely as an oxidant but functions as a specific signal activating a serine protease.

**Figure 9:**
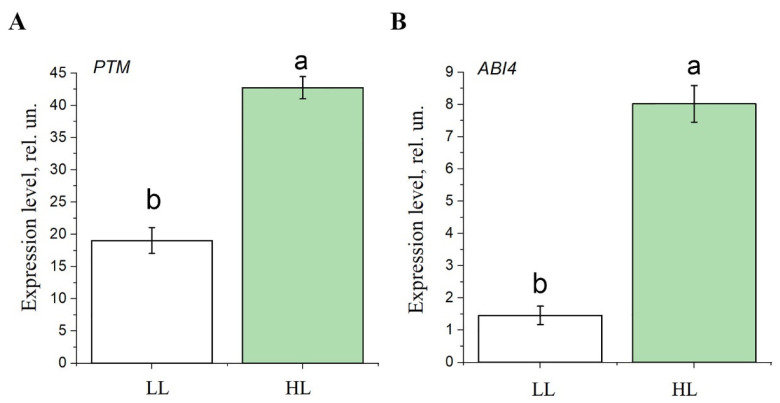
The effect of high light treatment on the expression level of genes encoding spinach transcription factors PTM (**A**) and ABI4 (**B**) in LL and after 4 days of constant HL in the leaves of *S. oleracea* plants. LL—plants grown at low light intensity (60–70 μmol quanta m^−2^ s^−1^, 16/8 h photoperiod), HL—plants transferred to high light conditions (HL, 500 μmol quanta m^−2^ s^−1^, 16/8 h photoperiod). Results are presented as the mean of three independent measurements. Error bars represent the standard error of the mean. Different letters above the bars indicate significant differences between variants with *p* < 0.05 as determined by ANOVA, followed by a Holm-Bonferroni test.

To verify whether the chloroplast-to-nucleus signal transduction mechanism leading to PS II antenna downsizing is similar in spinach and Arabidopsis, the expression of *ABI4* and *PTM* transcription factor genes in *S. oleracea* leaves was evaluated under low and high light conditions. After 4 days of HL, an increase in the mRNA level of the genes encoding these transcription factors was observed (Figure 9), as it was found for Arabidopsis genes (Figure 5). Since the spinach genome is not completely annotated, genes encoding chloroplast-envelope serine proteases have not yet been identified, and their expression could not be measured in the present study.

## 4. Discussion

The literature presents many studies addressing the issue of signal transduction from the chloroplast to the nucleus, but the molecular mechanisms of many signaling pathways have not yet been fully identified. Based on the data presented in Introduction, there are still several issues to be resolved for understanding the mechanism of the retrograde signaling for the PS II antenna downsizing. These are (i) what is the exact molecular mechanism of H_2_O_2_ involvement, (ii) which chloroplast-envelope protease participates in this process, and (iii) how this protease can be activated for initiating the signaling pathways studied. Our findings directly address these unresolved questions and provide one of the first mechanistic links connecting chloroplast-derived H_2_O_2_ with activation of a specific chloroplast-envelope protease and nuclear reprogramming of photosynthetic gene expression. This fills an important knowledge gap in understanding how redox changes at the thylakoid membrane are transmitted to the nucleus.

Plastid proteases have repeatedly been shown to be involved in plant signaling systems and metabolic processes in cells [29,61,62,63,64,65]. Proteases are found in thylakoids, chloroplast stroma, and in the chloroplast envelope [31,64]. In this work, the focus was given to the ASP protease, metalloprotease, located on the inner membrane of chloroplasts. ASP has already been ascribed a role as a participant in signaling [29]. The authors argue that ASP is a light-regulated protease essential for chloroplast development. Attention was also given to the serine protease SPPA1, which is located in both thylakoids and chloroplast envelope [31]. SPPA1 was first discovered in eubacteria, but it has also been found in viruses, archaea, and in chloroplasts of photoautotrophic eukaryotes [65]. While SPPA1 plays a role in antenna regulation in *Synechocystis* [30], its function in chloroplasts of higher plants is still not fully understood.

In the present work, we have observed that the addition of H_2_O_2_ at concentrations up to 100 μM increased total envelope protease activity, while the addition of a serine protease-specific inhibitor, PMSF, reduced this activity to the level observed in the absence of H_2_O_2_ (Figure 8). The data obtained indicate that H_2_O_2_ at low concentrations specifically activates serine protease activity. Therefore, we infer inclusion of a serine protease, presumably SPPA1, in the studied retrograde signaling pathway. These results provide evidence that H_2_O_2_, as a specific signaling messenger, exerts direct regulatory control over a certain enzymatic step that governs chloroplast-to-nucleus communication. Moreover, the data, showing upregulation of *SAPX* and *FC1* in high light, and no increase in *APX1*, *Cat2*, and *AOX1* (Figure 6D), particularly emphasize the functional contribution of chloroplast-derived H_2_O_2_ to the studied cellular adaptive response under the conditions examined. The possibility of activation of serine proteases by H_2_O_2_ has been previously shown. The activity of SBcas3.3 serine protease in *Escherichia coli*, which belongs to the S8A subfamily of serine proteases, increased upon addition of H_2_O_2_ at concentrations up to 10 g L^−1^, but decreased upon addition of H_2_O_2_ at a concentration of 50 g L^−1^ [46]. However, the effective concentrations for chloroplast membrane serine protease of higher plants and serine protease of *E. coli* appeared to be different. The activation of serine protease by H_2_O_2_ and, consequently, its inclusion in the retrograde pathway is consistent with the previously shown key role of serine protease in PTM processing. The use of the serine protease inhibitor has been shown to dramatically reduce PTM processing [27].

Given the established role of the metalloprotease ASP in plant growth and development, chloroplast biogenesis, and abiotic stress responses [29,65,66,67], its upregulation under high light conditions observed in our study likely reflects the activation of ASP-dependent adaptive mechanisms. This increase in *ASP* expression may represent an essential step of retrograde signaling triggered by chloroplast oxidative stress, thereby contributing to the coordination of cellular acclimation processes, although the molecular mechanisms of this protease involvement require further study [68].

Previously, we have shown that H_2_O_2_ molecules formed inside chloroplasts escape into the cytoplasm, bypassing intrachloroplast detoxification systems; the amount of H_2_O_2_ released into the cytoplasm depends on the light intensity, time of illumination, and activity of the chloroplast antioxidant system [44]. The role of chloroplast aquaporins, integral membrane proteins that form channels for small-sized neutral molecules [69], in the diffusion of H_2_O_2_ from the chloroplast into the cytoplasm was further proved [45]. The pathways of light-induced H_2_O_2_ formation in chloroplasts are described in detail in our reviews [39,43]. Our main discovery regarding H_2_O_2_ formation in chloroplasts, described in these reviews, was the fact that H_2_O_2_ can be formed not only in the chloroplast stroma due to the O_2_^•−^ dismutation reaction, but also directly on the surface of the thylakoid membrane (at the membrane/stroma phase boundary). The latter way of H_2_O_2_ formation proceeds owing to the reduction of O_2_^•−^ by PQH_2_ (see Introduction); this H_2_O_2_ was called the ‘membrane’ H_2_O_2_. In addition, we have shown that with increasing of light intensity, the enhancement in total H_2_O_2_ production in chloroplasts was precisely due to an increase in the formation of ‘membrane’ H_2_O_2_ [45]. That indicates a preferential contribution of ‘membrane’ H_2_O_2_ to the total amount of H_2_O_2_ diffusing outside the chloroplast into the cytoplasm. Importantly, while the involvement of ROS in retrograde signaling has been demonstrated, the identity of the specific ROS and their molecular targets remained unclear. Our results identify chloroplast-derived H_2_O_2_ and an envelope-localized serine protease as key functional components of this pathway.

**Figure 10:**
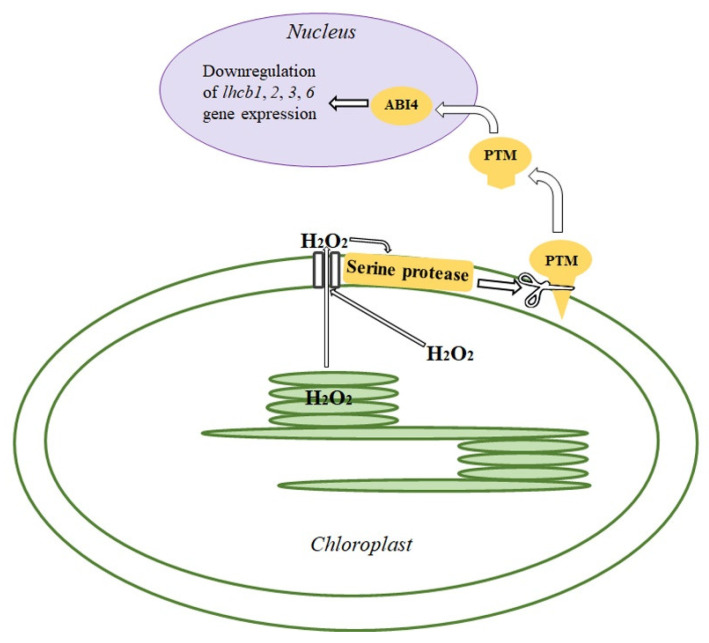
The hypothetical mechanism of the retrograde signaling proceeding for PS II antenna size downsizing. PTM, chloroplast envelope-associated homeodomain transcription factor; ABI4, nuclear transcription factor. H_2_O_2_ formed in chloroplasts diffuses through the chloroplast membrane and activates the serine protease. This protease converts PTM into its soluble form. The soluble PTM activates ABI4, which in turn binds to the promoter sequences of genes encoding Lhcb proteins in the nucleus, leading to inhibition of their gene expression.

Based on the data obtained in the present study, we conclude that H_2_O_2_ formed in chloroplasts, when diffusing through the chloroplast membrane, appears in close proximity to the serine protease, presumably SPPA1, of the chloroplast envelope and, by changing the activity of the protease, affects the transfer of PTM into the soluble form. PTM, after diffusing to the nucleus, activates the expression of the gene encoding transcription factor ABI4, which binds to the promoter sequences of *lhcb* genes and blocks the expression of *lhcb* genes, ultimately leading to PS II antenna downsizing (see Introduction). The mechanism of the retrograde signaling pathway of PS II antenna downsizing is shown in Figure 10.

It is logical that H_2_O_2_ at low concentrations activates the serine protease, since the amount of H_2_O_2_ that diffuses out of normally functioning chloroplasts corresponds to the low micromolar range [44]. The exact role of H_2_O_2_, depending on its concentrations, in the above-described retrograde signaling pathway may be as follows. H_2_O_2_, diffusing through the chloroplast membranes, at low concentrations (under conditions, e.g., of high light intensity, if the functioning of the chloroplast antioxidant system is not significantly inhibited), increases serine protease activity, leading to suppression of *lhcb* gene expression. At high H_2_O_2_ concentration (e.g., when the antioxidant system is inhibited under extreme stress conditions), this may, by contrast, lead to protease inactivation. As a consequence, acclimation changes in the size of the PS II antenna should not be observed, which are consistent with literature data [70].

In experiments, which were performed using plants adapted to a light intensity of 70 μmol quanta m^−2^ s^−1^ and plants exposed to HL (500 μmol quanta m^−2^ s^−1^) for 7–12 days, it was found that an increase in the mRNA level of ABI4 and PTM was observed under HL conditions (Figure 5). Thus, our data clearly demonstrate an activation of the PTM and ABI4-induced signaling pathway leading to the suppression of the biosynthesis of Lhcb proteins and, as a consequence, to a decrease in the PS II antenna size in HL. Previously, Koussevitzky et al. [57] and Moulin et al. [71] provided evidence of the involvement of ABI4 in ROS-mediated retrograde pathways. However, which type of ROS molecule is responsible for ABI4-mediated signaling for the PS II antenna size regulation is a key question that remains unanswered. Our findings suggest that the ABA-induced signaling pathway for the PS II antenna downsizing is based on effects associated with the generation of H_2_O_2_ rather than of other ROS. Such regulation can be triggered rapidly enough: already after 24 h of continuous HL H_2_O_2_ level was significantly enhanced, and the biosynthesis of ABI4, PTM, the serine protease, and Lhcb was altered (Figure 6). Under day/night period of HL treatment, these changes appeared later (Figure 1, Figure 2, Figure 3, Figure 4 and Figure 5).

Recent findings suggest that the regulation of PS II antenna size represents a widespread and evolutionarily conserved acclimatory response of the photosynthetic apparatus in higher plants. Importantly, antenna size adjustments are not only limited to light conditions changes but also are observed under a range of abiotic stress conditions, including drought, salinity, and temperature extremes. These stress factors commonly result in increased reduction of the PQ pool and elevated H_2_O_2_ levels in leaves [13,72,73]. Under these conditions, a consistent decrease in PS II antenna size has been documented, contributing to the mitigation of photooxidative stress through reduced light harvesting and improved redox balance. These observations support the idea that the pathway identified in our study is not limited to high-light acclimation, but represents a general regulatory module activated under multiple abiotic stress conditions, accompanied by elevated H_2_O_2_ production in chloroplasts. Interestingly, biotic factors may exert the opposite effect. For example, colonization of plants by the rhizosphere bacterium *Pseudomonas putida* BS3701 led to a decrease in both the PQ pool reduction and H_2_O_2_ accumulation, accompanied by an increase in PS II antenna size [74]. These findings further support the hypothesis that the redox state of the PQ pool and the intracellular H_2_O_2_ level act as central integrators of environmental signals, which regulate antenna size. Transcriptomic data from Genevestigator microarray analyses indicate that *ABI4* expression is also induced not only by HL, but by a broad spectrum of environmental stresses, including high and low temperature, osmotic stress, and salt exposure [75,76]. Importantly, ABI4 is also known to interact with hormonal signaling, including abscisic acid and ethylene, further linking chloroplast status with systemic stress adaptation and plant defense responses [58,77,78,79]. These observations suggest that ABI4 acts as an integrator of redox-derived (in particular, H_2_O_2_) and hormonal cues, coordinating nuclear gene expression changes in response to chloroplast stress. In this context, H_2_O_2_-mediated activation of chloroplast-envelope proteases may represent an upstream event that ultimately triggers ABI4-dependent transcriptional reprogramming. These observations align with the model proposed in this study (Figure 10), suggesting that the chloroplast-to-nucleus retrograde signaling pathway we describe, i.e., the pathway initiated by PQ pool over-reduction and H_2_O_2_ accumulation, and further mediated by SPPA1, PTM, and ABI4, can be a general regulatory mechanism activated under diverse stress conditions. Overall, our results highlight the central role of chloroplast-derived H_2_O_2_ as a signaling molecule that coordinates chloroplast function with nuclear gene expression.

## Figures and Tables

**Table 1:** Primers used for qRT-PCR. F is the “forward,” and R is the “reverse” primer.

Genes	Nucleotide Sequences of Primers	
*At2g32480* (NC_003071.7) (*Arabidopsis Serine Protease* (*ASP*) gene)	F	TGTGGGAAGGGAGTTTATGGGG
	R	GCTGCGAATTGGTAAAGCCC
*At1g73990* (NC_003070.9) (*Arabidopsis Signal Peptide Peptidase* (*SPPA1*) gene)	F	TCATTCTCGTGGTCTAATAGATGCTGTC
	R	CGTCGAGCAGTCCTTTTAATGTTCTG
*At5g35210* (NC_003076.8) (*Arabidopsis PTM* gene)	F	TGAAAAGGGTCTGAGATATTCATATAAGAGATCA
	R	GAGCACTCTGAGTCCAAGCAT
*At2g40220* (NC_003071.7) (*Arabidopsis ABI4* gene)	F	GTTGGAGATGGATCTTCGACCATTT
	R	TTGACCGACCTTAGGGATGCT
*At1g29930* (NC_003070.9) (*Arabidopsis lhcb1* gene)	F	AGCTCAAGAACGGAAGATTGG
	R	GCCAAATGGTCAGCAAGGTT
*At2g05070* (NC_003071.7) (*Arabidopsis lhcb2* gene)	F	GTCCATACCAGATGCTTTGGGGAG
	R	CTCACACTCTCTCTTCAATCCTTTCCTTTCAT
*At1g15820* (NC_003070.9) (*Arabidopsis lhcb6* gene)	F	GCGATGGCAGCGGTTCTTG
	R	CCATGGCGTTGCCCACTC
*At5g26030* (NC_003076.8) (*ATFC-I* (*Arabidopsis ferrochelatase I)* gene)	F	AGTTTCGTGAGTGAGCACATTGAG
	R	CATTTGGGTTTGACATTGCTTCTGCT
*At3g22370* (NC_003074.8) (*Arabidopsis AOX1* (*Alternative Oxidase 1*) gene)	F	AGAAGCGATCCATTCTTATACTGAGTTTCTC
	R	CGACCTTGGTAGTGAATATCAGATGC
*At1g07890* (NC_003070.9) (*Arabidopsis APX1* (*Ascorbate Peroxidase 1*) gene)	F	CTCTGGGACGATGCC
	R	GTATTTCTCGACCAAAGGAC
*At4g08390* (NC_003075.7) (*Arabidopsis SAPX* (*Stromal Ascorbate Peroxidase 1*) gene)	F	TTCTTTCAAGGTCTATGCTGA
	R	TAAGTCTGGTTGAGTAAATTATGTG
*At4g35090* (NC_003075.7) (*Arabidopsis Cat2* (*Peroxisome Catalase 2*) gene)	F	ACTTCAAGGAGCCTGGA
	R	AAGACTTATCAGCCTGAGAC
*At5g25760* (NC_003076.8) (*Arabidopsis Ubiquitin* gene)	F	TGCTTGGAGTCCTGCTTGGA
	R	TGTGCCATTGAATTGAACCCTCT
*LOC110783303* (NC_079490.1) (*Spinach PTM* gene)	F	AGAAAAACATCTTGGAAGAAAAGCAAATAAGC
	R	AAGTATCATCATCATCATCATCATCTACCCTT
*LOC110798630* (NC_079487.1) (*Spinach ABI4* gene)	F	GGAAGAATATCATCATAGTGCCAACTTTTG
	R	TAATTCTCCACCAAACTTTGATTTCCAAGG
*LOC110804113* (NC_079489.1) *Spinach Ubiquitin* gene	F	TTGTGTTGAGGCTAAGAGGTGG
	R	AAATCATTGCTGTGCCCACACTT

## Data Availability

The original contributions presented in this study are included in the article/Supplementary Materials. Further inquiries can be directed to the corresponding author.

## Supplementary Materials

The following supporting information can be downloaded at: https://www.mdpi.com/article/10.3390/antiox14121505/s1, Supplementary Figure S1. Immunoblot full membranes obtained after denaturing electrophoresis of the total protein leaf extracts with antibodies against Lhcb1 (A), Lhcb2 (B), Lhcb6 (C), D1 (D), and TOC-75 (E) proteins.

## Abbreviations

PETC	photosynthetic electron transport chain
PS II	photosystem II
PS I	photosystem I
PQ pool	plastoquinone pool
ROS	reactive oxygen species
H_2_O_2_	hydrogen peroxide
Lhcb proteins	light-harvesting complex proteins of photosystem II
PTM	a chloroplast envelope-bound plant homeodomain transcription factor
ABI4	abscisic acid insensitive 4 transcription factor
SPPA1	signal peptide peptidase
ASP	a light-regulated metalloprotease

## References

[1] Guskov A., Kern J., Gabdulkhakov A., Broser M., Zouni A., Saenger W. (2009). Cyanobacterial Photosystem II at 2.9-Å Resolution and the Role of Quinones, Lipids, Channels and Chloride. Nat. Struct. Mol. Biol..

[2] Umena Y., Kawakami K., Shen J.-R., Kamiya N. (2011). Crystal Structure of Oxygen-Evolving Photosystem II at a Resolution of 1.9 Å. Nature.

[3] Suga M., Akita F., Hirata K., Ueno G., Murakami H., Nakajima Y., Shimizu T., Yamashita K., Yamamoto M., Ago H. (2015). Native Structure of Photosystem II at 1.95 Å Resolution Viewed by Femtosecond X-Ray Pulses. Nature.

[4] Wei X., Su X., Cao P., Liu X., Chang W., Li M., Zhang X., Liu Z. (2016). Structure of Spinach Photosystem II–LHCII Supercomplex at 3.2 Å Resolution. Nature.

[5] Shi L.-X., Schröder W.P. (2004). The Low Molecular Mass Subunits of the Photosynthetic Supracomplex, Photosystem II. Biochim. Biophys. Acta (BBA)-Bioenerg..

[6] Boekema E.J., van Roon H., Calkoen F., Bassi R., Dekker J.P. (1999). Multiple Types of Association of Photosystem II and Its Light-Harvesting Antenna in Partially Solubilized Photosystem II Membranes. Biochemistry.

[7] Kouřil R., Wientjes E., Bultema J.B., Croce R., Boekema E.J. (2013). High-Light vs. Low-Light: Effect of Light Acclimation on Photosystem II Composition and Organization in *Arabidopsis thaliana*. Biochim. Biophys. Acta (BBA)-Bioenerg..

[8] Minagawa J. (2013). Dynamic Reorganization of Photosynthetic Supercomplexes during Environmental Acclimation of Photosynthesis. Front. Plant Sci..

[9] Damkjær J.T., Kereïche S., Johnson M.P., Kovacs L., Kiss A.Z., Boekema E.J., Ruban A.V., Horton P., Jansson S. (2009). The Photosystem II Light-Harvesting Protein Lhcb3 Affects the Macrostructure of Photosystem II and the Rate of State Transitions in Arabidopsis. Plant Cell.

[10] Jansson S. (1994). The Light-Harvesting Chlorophyll Ab-Binding Proteins. Biochim. Biophys. Acta (BBA)-Bioenerg..

[11] Aro E.M., McCaffery S., Anderson J.M. (1993). Photoinhibition and D1 Protein Degradation in Peas Acclimated to Different Growth Irradiances. Plant Physiol..

[12] Kale R., Hebert A.E., Frankel L.K., Sallans L., Bricker T.M., Pospíšil P. (2017). Amino Acid Oxidation of the D1 and D2 Proteins by Oxygen Radicals during Photoinhibition of Photosystem II. Proc. Natl. Acad. Sci. USA.

[13] Vetoshkina D., Balashov N., Ivanov B., Ashikhmin A., Borisova-Mubarakshina M. (2023). Light Harvesting Regulation: A Versatile Network of Key Components Operating under Various Stress Conditions in Higher Plants. Plant Physiol. Biochem..

[14] Anderson J.M. (1986). Photoregulation of the Composition, Function, and Structure of Thylakoid Membranes. Annu. Rev. Plant Physiol..

[15] Lindahl M., Yang D.-H., Andersson B. (1995). Regulatory Proteolysis of the Major Light-Harvesting Chlorophyll a/b Protein of Photosystem II by a Light-Induced Membrane-Associated Enzymic System. Eur. J. Biochem..

[16] Żelisko A., García-Lorenzo M., Jackowski G., Jansson S., Funk C. (2005). AtFtsH6 Is Involved in the Degradation of the Light-Harvesting Complex II during High-Light Acclimation and Senescence. Proc. Natl. Acad. Sci. USA.

[17] Wagner R., Aigner H., Pružinská A., Jänkänpää H.J., Jansson S., Funk C. (2011). Fitness Analyses of *Arabidopsis thaliana* Mutants Depleted of FtsH Metalloproteases and Characterization of Three FtsH6 Deletion Mutants Exposed to High Light Stress, Senescence and Chilling. New Phytol..

[18] Bailey S., Walters R.G., Jansson S., Horton P. (2001). Acclimation of *Arabidopsis thaliana* to the Light Environment: The Existence of Separate Low Light and High Light Responses. Planta.

[19] Ballottari M., Dall’Osto L., Morosinotto T., Bassi R. (2007). Contrasting Behavior of Higher Plant Photosystem I and II Antenna Systems during Acclimation *. J. Biol. Chem..

[20] Morosinotto T., Bassi R., Frigerio S., Finazzi G., Morris E., Barber J. (2006). Biochemical and Structural Analyses of a Higher Plant Photosystem II Supercomplex of a Photosystem I-Less Mutant of Barley. Consequences of a Chronic over-Reduction of the Plastoquinone Pool. FEBS J..

[21] Frigerio S., Campoli C., Zorzan S., Fantoni L.I., Crosatti C., Drepper F., Haehnel W., Cattivelli L., Morosinotto T., Bassi R. (2007). Photosynthetic Antenna Size in Higher Plants Is Controlled by the Plastoquinone Redox State at the Post-Transcriptional Rather than Transcriptional Level *. J. Biol. Chem..

[22] Borisova-Mubarakshina M.M., Vetoshkina D.V., Rudenko N.N., Shirshikova G.N., Fedorchuk T.P., Naydov I.A., Ivanov B.N. (2014). The Size of the Light-Harvesting Antenna of Higher Plant Photosystem Ii Is Regulated by Illumination Intensity through Transcription of Antenna Protein Genes. Biochem. Mosc..

[23] Yurina N.P., Odintsova M.S. (2019). Chloroplast Retrograde Signaling System. Russ. J. Plant Physiol..

[24] Woodson J.D., Chory J. (2008). Coordination of Gene Expression between Organellar and Nuclear Genomes. Nat. Rev. Genet..

[25] Chandrasekaran U., Luo X., Zhou W., Shu K. (2020). Multifaceted Signaling Networks Mediated by Abscisic Acid Insensitive 4. Plant Commun..

[26] Staneloni R.J., Rodriguez-Batiller M.J., Casal J.J. (2008). Abscisic Acid, High-Light, and Oxidative Stress Down-Regulate a Photosynthetic Gene via a Promoter Motif Not Involved in Phytochrome-Mediated Transcriptional Regulation. Mol. Plant.

[27] Sun X., Feng P., Xu X., Guo H., Ma J., Chi W., Lin R., Lu C., Zhang L. (2011). A Chloroplast Envelope-Bound PHD Transcription Factor Mediates Chloroplast Signals to the Nucleus. Nat. Commun..

[28] Adam Z. (2015). Plastid Intramembrane Proteolysis. Biochim. Biophys. Acta.

[29] Bölter B., Nada A., Fulgosi H., Soll J. (2006). A Chloroplastic Inner Envelope Membrane Protease Is Essential for Plant Development. FEBS Lett..

[30] Sokolenko A. (2005). SppA Peptidases: Family Diversity from Heterotrophic Bacteria to Photoautotrophic Eukaryotes. Physiol. Plant..

[31] Ferro M., Brugière S., Salvi D., Seigneurin-Berny D., Court M., Moyet L., Ramus C., Miras S., Mellal M., Le Gall S. (2010). AT_CHLORO, a Comprehensive Chloroplast Proteome Database with Subplastidial Localization and Curated Information on Envelope Proteins*. Mol. Cell. Proteom..

[32] Escoubas J.M., Lomas M., LaRoche J., Falkowski P.G. (1995). Light Intensity Regulation of Cab Gene Transcription Is Signaled by the Redox State of the Plastoquinone Pool. Proc. Natl. Acad. Sci. USA.

[33] Yang D.H., Andersson B., Aro E.M., Ohad I. (2001). The Redox State of the Plastoquinone Pool Controls the Level of the Light-Harvesting Chlorophyll a/b Binding Protein Complex II (LHC II) during Photoacclimation. Photosynth. Res..

[34] Chen Y.-B., Durnford D.G., Koblizek M., Falkowski P.G. (2004). Plastid Regulation of Lhcb1 Transcription in the Chlorophyte Alga Dunaliella Tertiolecta. Plant Physiol..

[35] Adamiec M., Drath M., Jackowski G. (2008). Redox State of Plastoquinone Pool Regulates Expression of *Arabidopsis thaliana* Genes in Response to Elevated Irradiance. Acta Biochim. Pol..

[36] Mubarakshina M., Khorobrykh S., Ivanov B. (2006). Oxygen Reduction in Chloroplast Thylakoids Results in Production of Hydrogen Peroxide inside the Membrane. Biochim. Biophys. Acta (BBA)-Bioenerg..

[37] Ivanov B., Mubarakshina M., Khorobrykh S. (2007). Kinetics of the Plastoquinone Pool Oxidation Following Illumination. FEBS Lett..

[38] Borisova-Mubarakshina M.M., Naydov I.A., Ivanov B.N. (2018). Oxidation of the Plastoquinone Pool in Chloroplast Thylakoid Membranes by Superoxide Anion Radicals. FEBS Lett..

[39] Kozuleva M.A., Ivanov B.N., Vetoshkina D.V., Borisova-Mubarakshina M.M. (2020). Minimizing an Electron Flow to Molecular Oxygen in Photosynthetic Electron Transfer Chain: An Evolutionary View. Front. Plant Sci..

[40] Sewelam N., Jaspert N., Van Der Kelen K., Tognetti V.B., Schmitz J., Frerigmann H., Stahl E., Zeier J., Van Breusegem F., Maurino V.G. (2014). Spatial H_2_O_2_ Signaling Specificity: H_2_O_2_ from Chloroplasts and Peroxisomes Modulates the Plant Transcriptome Differentially. Mol. Plant.

[41] Borisova-Mubarakshina M., Ivanov B. (2010). The Production and Scavenging of Reactive Oxygen Species in the Plastoquinone Pool of Chloroplast Thylakoid Membranes. Physiol. Plant..

[42] Borisova-Mubarakshina M.M., Ivanov B.N., Vetoshkina D.V., Lubimov V.Y., Fedorchuk T.P., Naydov I.A., Kozuleva M.A., Rudenko N.N., Dall’Osto L., Cazzaniga S. (2015). Long-Term Acclimatory Response to Excess Excitation Energy: Evidence for a Role of Hydrogen Peroxide in the Regulation of Photosystem II Antenna Size. J. Exp. Bot..

[43] Borisova-Mubarakshina M.M., Vetoshkina D.V., Ivanov B.N. (2019). Antioxidant and Signaling Functions of the Plastoquinone Pool in Higher Plants. Physiol. Plant.

[44] Mubarakshina M.M., Ivanov B.N., Naydov I.A., Hillier W., Badger M.R., Krieger-Liszkay A. (2010). Production and Diffusion of Chloroplastic H_2_O_2_ and Its Implication to Signalling. J. Exp. Bot..

[45] Borisova M.M.M., Kozuleva M.A., Rudenko N.N., Naydov I.A., Klenina I.B., Ivanov B.N. (2012). Photosynthetic Electron Flow to Oxygen and Diffusion of Hydrogen Peroxide through the Chloroplast Envelope via Aquaporins. Biochim. Biophys. Acta (BBA)-Bioenerg..

[46] Biver S., Portetelle D., Vandenbol M. (2013). Characterization of a New Oxidant-Stable Serine Protease Isolated by Functional Metagenomics. SpringerPlus.

[47] Tóth S.Z., Nagy V., Puthur J.T., Kovács L., Garab G. (2011). The Physiological Role of Ascorbate as Photosystem II Electron Donor: Protection against Photoinactivation in Heat-Stressed Leaves. Plant Physiol..

[48] Kalaji H.M., Schansker G., Ladle R.J., Goltsev V., Bosa K., Allakhverdiev S.I., Brestic M., Bussotti F., Calatayud A., Dąbrowski P. (2014). Frequently Asked Questions about in Vivo Chlorophyll Fluorescence: Practical Issues. Photosynth. Res..

[49] Cormier M.J., Prichard P.M. (1968). An Investigation of the Mechanism of the Luminescent Peroxidation of Luminol by Stopped Flow Techniques. J. Biol. Chem..

[50] Douce R., Holtz R.B., Benson A.A. (1973). Isolation and Properties of the Envelope of Spinach Chloroplasts. J. Biol. Chem..

[51] Conlon H.E., Salter M.G. (2007). Plant Protein Extraction. Methods Mol. Biol..

[52] Lichtenthaler H.K. (1987). Chlorophylls and Carotenoids: Pigments of Photosynthetic Biomembranes. Methods in Enzymology.

[53] Schägger H., von Jagow G. (1987). Tricine-Sodium Dodecyl Sulfate-Polyacrylamide Gel Electrophoresis for the Separation of Proteins in the Range from 1 to 100 kDa. Anal. Biochem..

[54] Strasser R.J., Srivastava A., Tsimilli-Michael M. (2000). The Fluorescence Transient as a Tool to Characterize and Screen Photosynthetic Samples. Probing Photosynthesis: Mechanisms, Regulation and Adaptation.

[55] Schöttler M.A., Tóth S.Z. (2014). Photosynthetic Complex Stoichiometry Dynamics in Higher Plants: Environmental Acclimation and Photosynthetic Flux Control. Front. Plant Sci..

[56] Pfannschmidt T., Nilsson A., Allen J.F. (1999). Photosynthetic Control of Chloroplast Gene Expression. Nature.

[57] Koussevitzky S., Nott A., Mockler T.C., Hong F., Sachetto-Martins G., Surpin M., Lim J., Mittler R., Chory J. (2007). Signals from Chloroplasts Converge to Regulate Nuclear Gene Expression. Science.

[58] León P., Gregorio J., Cordoba E. (2013). ABI4 and Its Role in Chloroplast Retrograde Communication. Front. Plant Sci..

[59] Exposito-Rodriguez M., Laissue P.P., Yvon-Durocher G., Smirnoff N., Mullineaux P.M. (2017). Photosynthesis-Dependent H_2_O_2_ Transfer from Chloroplasts to Nuclei Provides a High-Light Signalling Mechanism. Nat. Commun..

[60] Tranel P.J., Froehlich J., Goyal A., Keegstra K. (1995). A Component of the Chloroplastic Protein Import Apparatus Is Targeted to the Outer Envelope Membrane via a Novel Pathway. EMBO J..

[61] Abraham L.D., Breuil C. (1996). Isolation and Characterization of a Subtilisin-like Serine Proteinase Secreted by the Sap-Staining Fungus *Ophiostoma piceae*. Enzym. Microb. Technol..

[62] Yano A., Suzuki K., Shinshi H. (1999). A Signaling Pathway, Independent of the Oxidative Burst, That Leads to Hypersensitive Cell Death in Cultured Tobacco Cells Includes a Serine Protease. Plant J..

[63] Antão C.M., Malcata F.X. (2005). Plant Serine Proteases: Biochemical, Physiological and Molecular Features. Plant Physiol. Biochem..

[64] Sakamoto W. (2006). Protein Degradation Machineries in Plastids. Annu. Rev. Plant Biol..

[65] Wetzel C.M., Harmacek L.D., Yuan L.H., Wopereis J.L.M., Chubb R., Turini P. (2009). Loss of Chloroplast Protease SPPA Function Alters High Light Acclimation Processes in *Arabidopsis thaliana* L. (Heynh.). J. Exp. Bot..

[66] Adamiec M., Ciesielska M., Zalaś P., Luciński R. (2017). *Arabidopsis thaliana* Intramembrane Proteases. Acta Physiol. Plant.

[67] Luciński R., Adamiec M. (2023). The Role of Plant Proteases in the Response of Plants to Abiotic Stress Factors. Front. Plant Physiol..

[68] Pojidaeva E.S., Voloshin R.A., Gorshkova D.S., Fedorova E.E., Piotrovsky M.S., Kusnetsov V.V. (2025). Chloroplast Members of the S2P Proteases Family, AraSP and AtS2P2, May Have a Functional Relationship in Promoting Plant Growth. Russ. J. Plant Physiol..

[69] Henzler T., Steudle E. (2000). Transport and Metabolic Degradation of Hydrogen Peroxide in Chara Corallina: Model Calculations and Measurements with the Pressure Probe Suggest Transport of H_2_O_2_ across Water Channels. J. Exp. Bot..

[70] Ksas B., Becuwe N., Chevalier A., Havaux M. (2015). Plant Tolerance to Excess Light Energy and Photooxidative Damage Relies on Plastoquinone Biosynthesis. Sci. Rep..

[71] Moulin M., McCormac A.C., Terry M.J., Smith A.G. (2008). Tetrapyrrole Profiling in Arabidopsis Seedlings Reveals That Retrograde Plastid Nuclear Signaling Is Not Due to Mg-Protoporphyrin IX Accumulation. Proc. Natl. Acad. Sci. USA.

[72] Tikkanen M., Aro E.-M. (2014). Integrative Regulatory Network of Plant Thylakoid Energy Transduction. Trends Plant Sci..

[73] Borisova-Mubarakshina M.M., Vetoshkina D.V., Naydov I.A., Rudenko N.N., Zhurikova E.M., Balashov N.V., Ignatova L.K., Fedorchuk T.P., Ivanov B.N. (2020). Regulation of the Size of Photosystem II Light Harvesting Antenna Represents a Universal Mechanism of Higher Plant Acclimation to Stress Conditions. Funct. Plant Biol..

[74] Vetoshkina D.V., Pozdnyakova-Filatova I.Y., Zhurikova E.M., Frolova A.A., Naydov I.A., Ivanov B.N., Borisova-Mubarakshina M.M. (2019). The Increase in Adaptive Capacity to High Illumination of Barley Plants Colonized by Rhizobacteria *P. Putida* BS3701. Appl. Biochem. Microbiol..

[75] Zimmermann P., Hirsch-Hoffmann M., Hennig L., Gruissem W. (2004). GENEVESTIGATOR. Arabidopsis Microarray Database and Analysis Toolbox. Plant Physiol..

[76] Yoshida T., Mogami J., Yamaguchi-Shinozaki K. (2014). ABA-Dependent and ABA-Independent Signaling in Response to Osmotic Stress in Plants. Curr. Opin. Plant Biol..

[77] Signora L., De Smet I., Foyer C.H., Zhang H. (2001). ABA Plays a Central Role in Mediating the Regulatory Effects of Nitrate on Root Branching in Arabidopsis. Plant J..

[78] Kaliff M., Staal J., Myrenås M., Dixelius C. (2007). ABA Is Required for Leptosphaeria Maculans Resistance via ABI1- and ABI4-Dependent Signaling. Mol. Plant Microbe Interact..

[79] Shkolnik-Inbar D., Bar-Zvi D. (2010). ABI4 Mediates Abscisic Acid and Cytokinin Inhibition of Lateral Root Formation by Reducing Polar Auxin Transport in Arabidopsis. Plant Cell.

