# Supplementary material for: Revealing Missing Links in the Downsizing of the Photosystem II Antenna in Higher Plants Under Stress Conditions
Source: Antioxidants (Basel). 2025 Dec 15;14(12):1505. doi: 10.3390/antiox14121505 (PMC12729535; doi:10.3390/antiox14121505)
Supplement: Supplementary file 1 [file antioxidants-14-01505-s001.zip › antioxidants-3987386-supplementary.pdf]

Full unedited gel for Figure 2 (A)

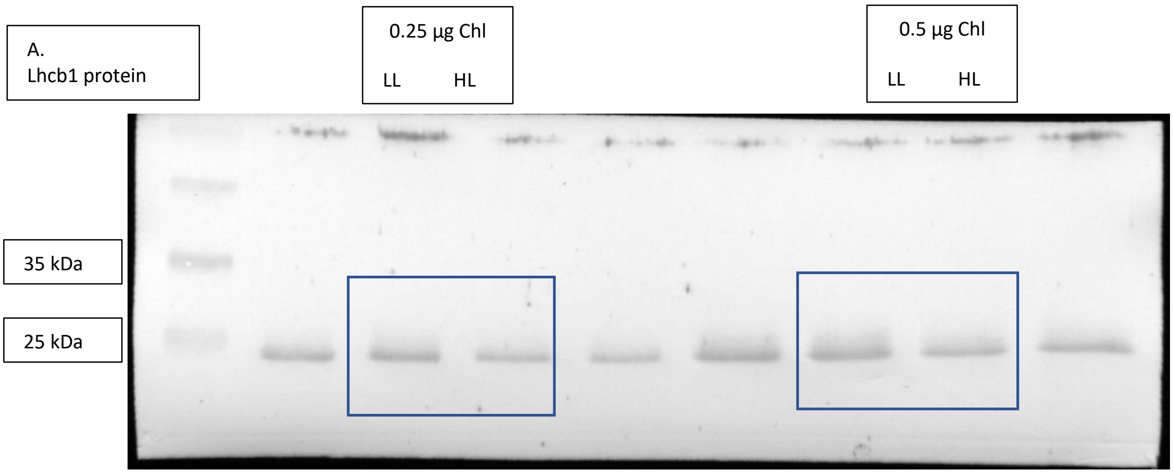

Lanes presented in the manuscript are highlighted with rectangles.

Full unedited gel for Figure 2 (B)

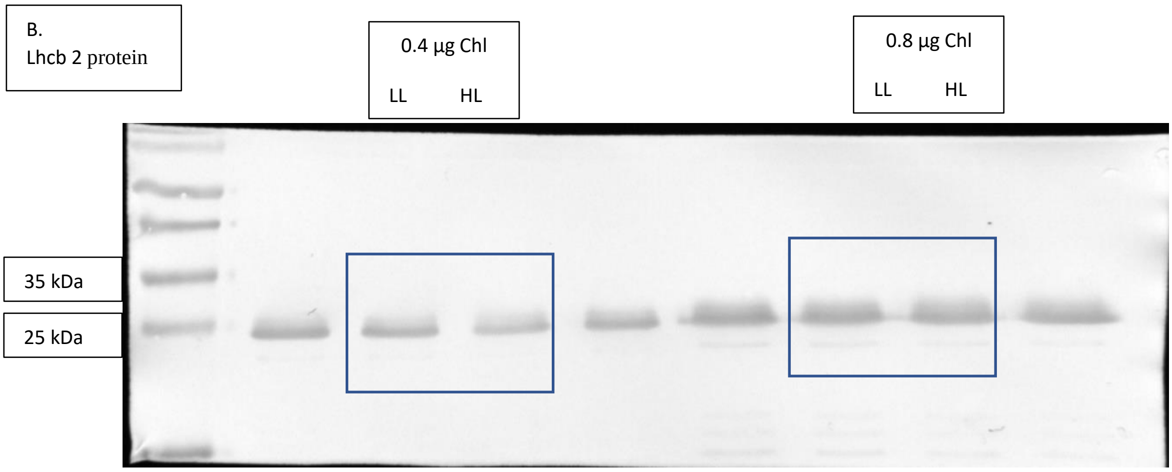

Lanes presented in the manuscript are highlighted with rectangles.

Full unedited gel for Figure 2 (C)

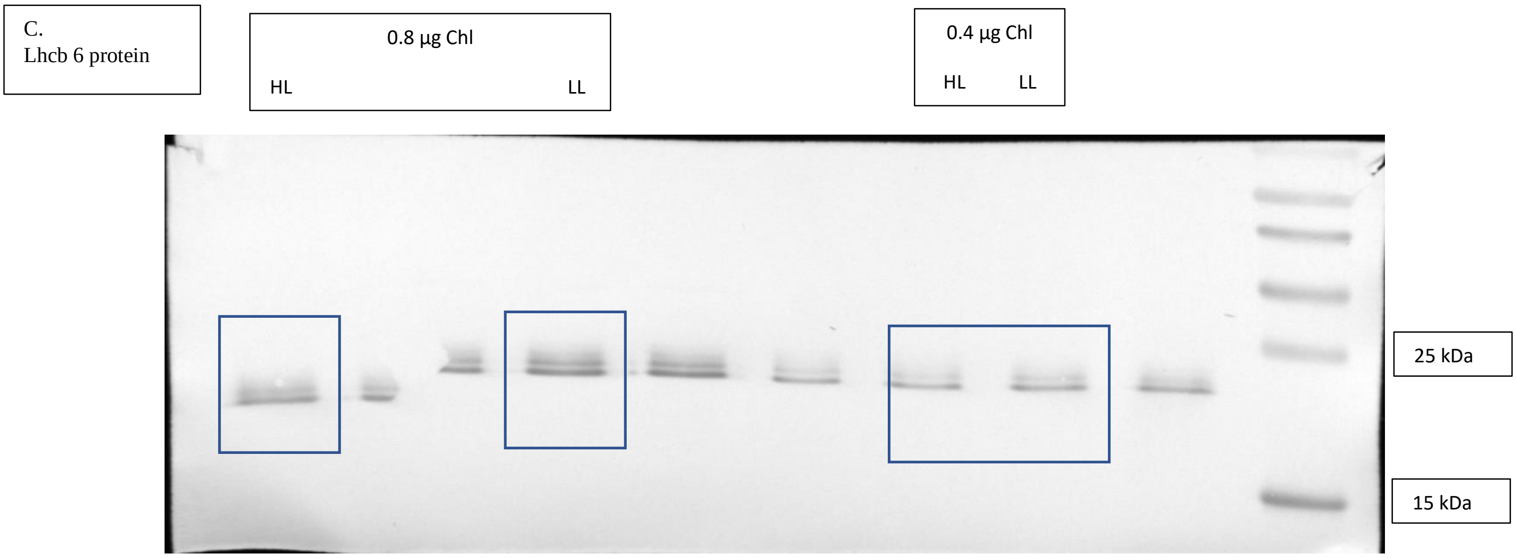

Lanes presented in the manuscript are highlighted with rectangles.

Full unedited gel for Figure 2 (D)

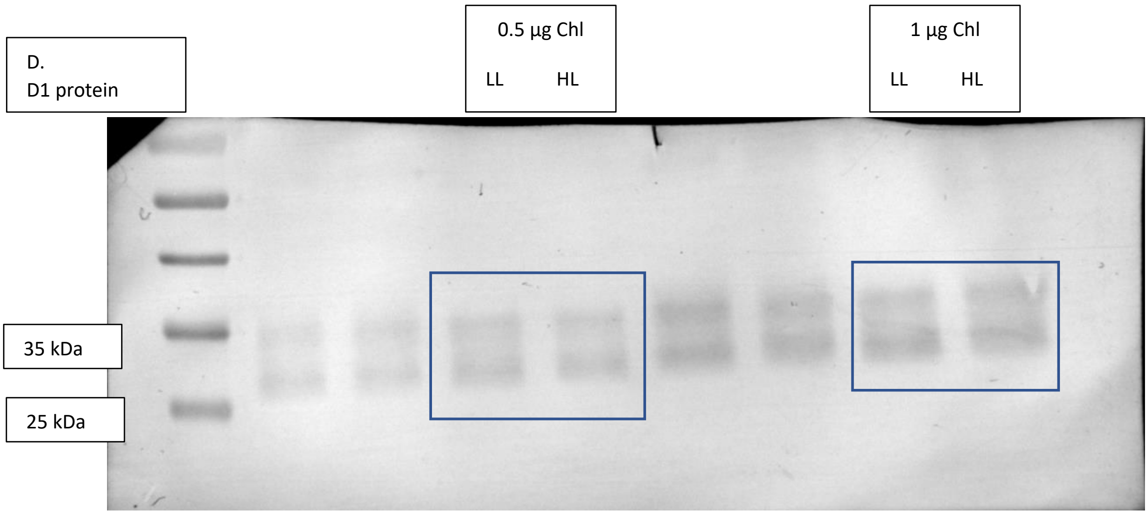

Lanes presented in the manuscript are highlighted with rectangles.

Full unedited gel for Figure 7

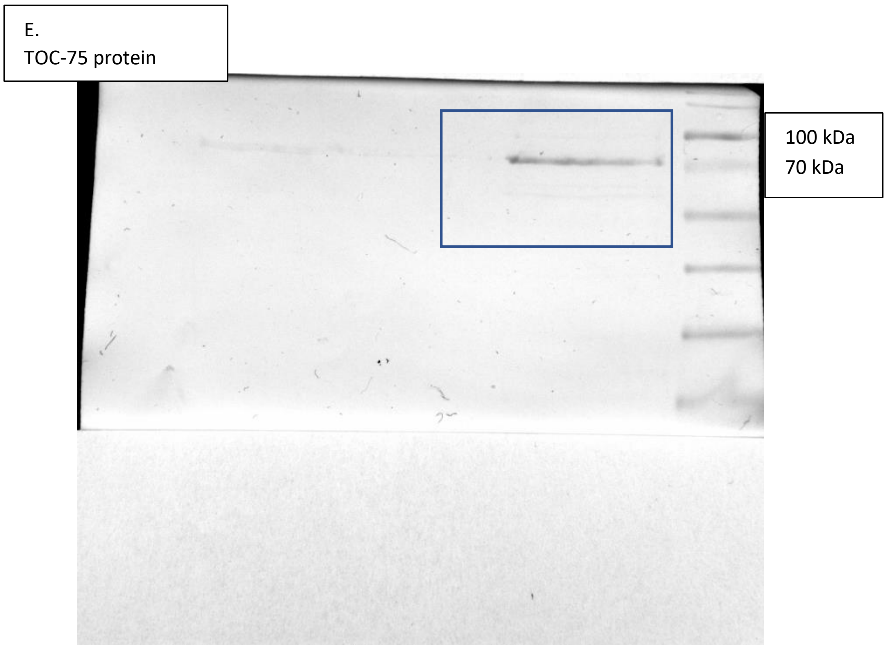

Lanes presented in the manuscript are highlighted with rectangles.

Supplementary Figure S1. Immunoblot full membranes obtained after denaturing electrophoresis of the total protein leaf extracts with antibodies against Lhcb1 (A), Lhcb2 (B), Lhcb6 (C), D1 (D) and TOC-75 (E) proteins.
